# Hesperetin-Loaded PLGA Nanoparticles Ameliorate Cisplatin-Induced Oxidative Stress and Testicular Dysfunction in Rats: Association with NRF2/HO-1, NF-κB, and ACSL4/GPX4/SLC7A11 Signaling Modulation

**DOI:** 10.3390/antiox15081007

**Published:** 2026-08-13

**Authors:** Mohammed A. Akeel, Ekramy M. Elmorsy, Aly A. M. Shaalan, Fahad Alshammari, Ezzat A. Ismail, Gehad E. Elshopakey, Manal S. Fawzy, Shaimaa A. Shehata

**Affiliations:** 1 Department of Human Anatomy, Faculty of Medicine, Jazan University, Jazan 45142, Saudi Arabia; m.akeel@jazanu.edu.sa (M.A.A.); ashaalan@jazanu.edu.sa (A.A.M.S.); 2 Center for Health Research, Northern Border University, Arar 73213, Saudi Arabia; ekramy.elmorsy@nbu.edu.sa; 3 Department of Histology and Cell Biology, Faculty of Medicine, Suez Canal University, Ismailia 41522, Egypt; 4 Department of Biology, College of Science, Jouf University, Sakaka 72341, Aljouf, Saudi Arabia; fahadm@ju.edu.sa; 5 Department of Urology, Faculty of Medicine, Suez Canal University, Ismailia 41522, Egypt; boezzat@med.suez.edu.eg; 6 Department of Clinical Pathology, Faculty of Veterinary Medicine, Mansoura University, Mansoura 35516, Egypt; gehadelshopakey@mans.edu.eg; 7 Department of Forensic Medicine and Clinical Toxicology, Faculty of Medicine, Suez Canal University, Ismailia 41522, Egypt; shaimaa_shehata@med.suez.edu.eg

**Keywords:** hesperetin, PLGA nanoparticles, cisplatin, oxidative stress, NRF2/HO-1, NF-κB, ferroptosis, male reproductive health

## Abstract

Background: Cisplatin is a widely used chemotherapeutic agent whose gonadotoxic effects, largely driven by oxidative stress and inflammation, pose a major threat to male reproductive health. This study evaluated whether hesperetin (HES) and hesperetin-loaded poly (lactic-co-glycolic acid) nanoparticles (HES-PLGA-NPs) can protect against cisplatin-induced testicular dysfunction in adult male Sprague Dawley rats. Methods: Sixty rats were randomly assigned to six groups: control, HES, HES-PLGA-NPs, cisplatin (CIS), CIS + HES, and CIS + HES-PLGA-NPs. CIS (7.5 mg/kg/week, i.p.) was given intraperitoneally for four weeks, while HES and nano-HES (50 mg/kg/day, p.o.) were orally administered concurrently. Reproductive hormones, testicular weight, sperm parameters, oxidative stress and antioxidant markers, NRF2/HO-1 and NF-κB signaling, apoptotic indices, ferroptosis-related markers, histopathology, and GPX4/ACSL4 immunoexpression were assessed. Results: CIS caused marked reproductive dysfunction, including reduced testosterone, FSH, and LH, decreased testicular weight, and impaired sperm quality. These changes were associated with severe oxidative and nitrosative stress (↑ MDA, NO, 8-OHdG), depletion of antioxidant defenses (↓ SOD, CAT, GSH), reduced NRF2/HO-1, elevated NF-κB activity and pro-inflammatory cytokines, apoptosis-related changes (↑ Bax, caspase-3; ↓ Bcl-2), and ferroptosis-associated alterations (↑ Fe^2+^, TFR1, lipid ROS, ACSL4, 4-HNE; ↓ GPX4, SLC7A11). Both HES and HES-PLGA-NPs were associated with significant amelioration of these alterations, but the nanoformulation showed more pronounced protection, normalizing several oxidative and ferroptosis-associated markers toward control levels and more effectively preserving seminiferous tubule architecture and spermatogenesis. Conclusions: HES-PLGA-NPs were associated with multi-mechanistic protection against cisplatin-induced oxidative, inflammatory, apoptotic, and ferroptosis-associated damage in the testes, and may represent a promising nanoantioxidant candidate warranting further investigation for preserving male reproductive function during chemotherapy.

## 1. Introduction

Cisplatin remains a cornerstone chemotherapeutic agent and continues to play a central role in the management of a wide range of malignancies [1,2]. It is routinely used in the treatment of colorectal, hematological, lung, ovarian, head and neck, and testicular cancers, among others [3]. Its potent anticancer activity is largely attributed to its ability to form DNA adducts and inter- and intra-strand cross-links, thereby disrupting DNA replication and transcription in rapidly proliferating tumor cells [4,5]. These lesions trigger S-phase arrest and activate both apoptotic and necrotic cell death pathways [1]. In addition, cisplatin promotes the generation of reactive oxygen species (ROS) and induces oxidative stress, a mechanism that contributes to its cytotoxic action but also underlies much of its off-target toxicity in normal tissues [6,7].

Despite its unquestionable value in oncology, the clinical utility of cisplatin is restricted by dose-limiting adverse effects, with the male reproductive system being particularly vulnerable [8]. Testicular tissue is highly sensitive because of the rapid turnover of germ cells and their susceptibility to redox imbalance [9]. Cisplatin-induced testicular toxicity typically manifests as degeneration of seminiferous tubules, disruption of spermatogenesis, deterioration in sperm count, motility and morphology, and dysregulation of reproductive hormones, including testosterone, FSH, and LH [4,10]. At the mechanistic level, these deleterious effects have been linked to excessive oxidative stress, persistent inflammation, mitochondrial dysfunction, and activation of intrinsic apoptotic signaling [11,12,13]. More recently, ferroptosis has emerged as an additional regulated cell death pathway involved in cisplatin-induced testicular injury [14]. Ferroptosis is characterized by iron-dependent lipid peroxidation, suppression of key antioxidant defenses such as GPX4, and accumulation of Fe^2+^ and lipid ROS [15,16,17]. This process is further associated with elevated levels of 4-HNE and dysregulated expression of crucial regulators, including SLC7A11 and ACSL4 [18]. By coupling oxidative stress with membrane lipid damage, ferroptosis contributes to germ cell loss and defective spermatogenesis in cisplatin-treated testes [14].

Hesperetin (HES), a citrus-derived flavanone, has attracted attention as a promising candidate because of its broad pharmacological profile [19]. It exerts pronounced antioxidant, anti-inflammatory, and anti-apoptotic effects, largely through direct free-radical scavenging, inhibition of lipid peroxidation, and modulation of redox-sensitive signaling pathways [19,20,21,22]. Experimental data indicate that HES can protect the testes by limiting oxidative damage, dampening inflammatory responses, and restraining apoptotic cell death [21,23]. However, the translational potential of HES is hampered by intrinsic biopharmaceutical limitations, including poor aqueous solubility, lipophilicity, low oral bioavailability, and consequently suboptimal systemic exposure [24].

Nanotechnology-based delivery systems have been proposed as an effective strategy to overcome these drawbacks and enhance the therapeutic utility of hydrophobic phytochemicals. Encapsulation within nanocarriers can improve solubility and stability, reduce premature degradation, and enable sustained and controlled drug release at target sites [25]. Among these platforms, poly (lactic-co-glycolic acid) (PLGA) nanoparticles are widely used owing to their biocompatibility, biodegradability, and regulatory approval for multiple biomedical applications [26]. PLGA-based nanocarriers efficiently encapsulate hydrophobic natural products, thereby improving pharmacokinetics, enhancing cellular uptake, and increasing therapeutic efficacy compared with free compounds [24,27]. Loading HES into PLGA nanoparticles is therefore expected to circumvent its physicochemical constraints and potentiate its biological activity in vivo [24,28]. What remains unresolved, however, is whether such nanoencapsulation confers measurable advantages in a clinically relevant model of testicular toxicity and how it influences the complex network of oxidative, inflammatory, apoptotic, and ferroptotic pathways implicated in cisplatin-induced damage.

In this context, several related studies help define the existing knowledge base. Kaya et al. reported that hesperidin, the glycoside form of hesperetin, attenuates cisplatin-induced testicular and spermatological damage, mainly by reducing oxidative stress in rats [29]. That study demonstrated the protective potential of a citrus flavonoid in cisplatin-associated reproductive toxicity, but it used free hesperidin, focused on conventional oxidative and sperm parameters, and did not explore nanoparticle-based delivery or ferroptosis. Duranoğlu et al. successfully developed and optimized hesperetin-loaded PLGA nanoparticles, confirming favorable encapsulation efficiency, particle stability, and biocompatibility in vitro [24]. Their work, however, was restricted to formulation and physicochemical characterization, without in vivo organ toxicity evaluation or a reproductive model. Furthermore, Samie et al. showed that free hesperetin attenuates streptozotocin-induced diabetic testicular damage by inhibiting oxidative stress, inflammation, and apoptosis, and by improving sperm quality and testosterone levels [23]. Although this study highlights the intrinsic testicular protective properties of hesperetin, it addressed a chronic hyperglycemic model, did not use a nanoencapsulated formulation, and did not examine ferroptosis-related markers. Taken together, these reports support the biological plausibility of hesperetin as a testicular protectant and demonstrate that PLGA-based hesperetin nanoparticles are feasible, yet none has combined hesperetin nanoencapsulation with in vivo cisplatin-induced testicular toxicity or interrogated ferroptotic signaling.

The present study was designed to address this gap by evaluating the protective effects of hesperetin-loaded PLGA nanoparticles (HES-PLGA-NPs) against cisplatin-induced testicular damage in rats, in direct comparison with free HES. The experimental protocol included a comprehensive assessment of reproductive outcomes, hormonal profile, sperm characteristics, and testicular histology to gauge functional recovery. Beyond these functional endpoints, we examined a multi-pathway mechanistic axis linking NRF2/HO-1-mediated antioxidant defense, NF-κB-driven inflammatory signaling, and the ferroptosis regulators ACSL4, GPX4, and SLC7A11. To our knowledge, this is the first study to bring nanoparticle-enhanced hesperetin delivery, in vivo testicular protection, and ferroptotic signaling together within a single experimental framework, distinguishing it from prior work that examined these elements only in isolation.

## 2. Materials and Methods

### 2.1. Synthesis of Hesperetin-Loaded PLGA Nanoparticles (HES-PLGA-NPs)

Hesperetin-loaded PLGA nanoparticles were prepared by a single emulsion–solvent evaporation method [24] with minor optimization. Briefly, hesperetin was dissolved in 1 mL of acetone, while PLGA was dissolved separately in 1 mL of dichloromethane (DCM). The two organic solutions were then mixed to obtain a homogeneous organic phase. This organic phase was slowly added to an aqueous polyvinyl alcohol (PVA) solution under continuous stirring to form a coarse emulsion, which was subsequently sonicated to enhance emulsification and reduce droplet size. The resulting emulsion was gradually introduced into 0.1% PVA solution using a peristaltic pump to ensure uniform dispersion and controlled droplet formation. Organic solvents were allowed to evaporate under magnetic stirring at 700 rpm at room temperature overnight, yielding a stable nanosuspension.

The nanosuspension was centrifuged at 14,000 rpm for 40 min at 4 °C (Hettich Universal 32R, Tuttlingen, Germany), and the pellet was collected. The precipitated nanoparticles were washed three times with 35 mL of distilled water to remove residual PVA and unencapsulated HES. Supernatants from the wash steps were retained to determine encapsulation efficiency.

### 2.2. Physicochemical Characterization

The hydrodynamic diameter, polydispersity index (PDI), and zeta potential of HES-PLGA-NPs were determined by dynamic light scattering using a Zetasizer Nano ZS (Malvern Instruments, Malvern, UK). Measurements were performed in triplicate after appropriate dilution of nanosuspensions with deionized water to avoid multiple scattering. The Z-average diameter and PDI were recorded for each sample.

Nanoparticle morphology was evaluated by transmission electron microscopy (TEM; JEOL 2100, Tokyo, Japan) operated at 160 kV. A drop of diluted nanosuspension was placed on a carbon-coated copper grid, allowed to dry at room temperature, and imaged to visualize particle shape and surface characteristics. Encapsulation efficiency (*EE*, %) was calculated using the following equation:

$$EE\;\left(\%\right)=\frac{\left(initial\;HES\;-free\;HES\right)}{initial\;HES}\;\times 100$$

where free *HES* in the supernatant was quantified spectrophotometrically.

### 2.3. In Vitro Drug Release

The in vitro release profile of HES from PLGA nanoparticles was investigated using a dialysis membrane diffusion method. A known quantity of HES-PLGA-NPs equivalent to a defined amount of HES was dispersed in phosphate-buffered saline (PBS, pH 7.4) and placed into a dialysis bag with an appropriate molecular weight cut-off. The dialysis bag was then immersed in PBS (pH 7.4) at 37 °C under gentle shaking.

At pre-defined time intervals, aliquots were withdrawn from the release medium and immediately replaced with equal volumes of fresh pre-warmed PBS to maintain sink conditions. The amount of HES released in each sample was determined spectrophotometrically at the characteristic absorption wavelength using a calibration curve prepared from standard HES solutions. The cumulative percentage of HES released was plotted against time.

### 2.4. Fourier Transform Infrared Spectroscopy (FTIR)

FTIR analysis was performed to evaluate potential interactions between HES and the PLGA matrix and to ensure successful encapsulation. Separate samples of pure HES, blank PLGA nanoparticles, and HES-PLGA-NPs were analyzed. Spectra were recorded in the range of 4000–400 cm^−1^ using an FTIR spectrometer (PerkinElmer, Waltham, MA, USA). The characteristic absorption bands of HES were compared before and after encapsulation. Any shift in peak position, change in intensity, or disappearance of specific bands was examined to infer drug–polymer interactions and HES entrapment within the PLGA network.

### 2.5. Experimental Animals and Study Design

A total of 60 adult male Sprague Dawley rats (270–310 g) were obtained from the Medical Experimental Research Center (MERC), Faculty of Medicine, Mansoura University, Egypt. Animals were acclimatized for two weeks and housed under standard laboratory conditions (25 ± 2 °C, 45% humidity, 12 h light/dark cycle) with free access to a standard rodent diet and water, in accordance with ARRIVE 2.0 guidelines [30].

The sub-acute oral toxicity of HES and HES-PLGA-NPs was evaluated in a preliminary study conducted in accordance with OECD guidelines. All procedures were approved by the institutional animal care and use committee (i.e., Faculty of Veterinary Medicine, Mansoura University, Egypt; VM.R.26.07.306) and complied with international standards for the care and use of animals.

HES (≥98% purity) and reagents were obtained from commercial suppliers. The oral dose of HES (50 mg/kg/day) was selected based on previous reports demonstrating efficacy and safety in rodent models [31]. HES-PLGA-NPs were administered at an equivalent HES dose (50 mg/kg/day) to enable direct comparison with the free compound and to evaluate the influence of PLGA nanoencapsulation on therapeutic efficacy. Cisplatin was administered at 7.5 mg/kg/week intraperitoneally, a well-established regimen that reliably induces testicular toxicity while maintaining acceptable animal survival in experimental models [32].

Animals were randomly allocated into six groups (n = 10 per group) and treated for four consecutive weeks:

• Control group: received normal saline and served as the baseline reference.
• HES group: received oral free hesperetin (50 mg/kg/day) to evaluate the effect of HES alone.
• HES-PLGA-NPs group: received an equivalent oral dose of hesperetin encapsulated in PLGA nanoparticles (50 mg/kg/day) to assess the effect of the nanoformulation alone.
• Cis group: received cisplatin (7.5 mg/kg/week, i.p.) and served as the untreated toxicity model.
• Cis + HES group: received cisplatin (7.5 mg/kg/week, i.p.) plus oral HES (50 mg/kg/day).
• Cis + HES-PLGA-NPs group: received cisplatin (7.5 mg/kg/week, i.p.) plus oral HES-PLGA-NPs (50 mg/kg/day).

Treatments were administered in a consistent order each day to minimize circadian and handling-related variability. The primary outcome measures were sperm parameters, serum reproductive hormones, testicular oxidative stress/antioxidant markers, inflammatory, apoptotic, and ferroptosis-related indices, and histopathology. No animals met pre-defined humane endpoints or required early euthanasia.

### 2.6. Sample Collection and Processing

At the end of the experiment, rats were fasted overnight and euthanized under isoflurane anesthesia. Blood was collected from the retro-orbital venous plexus. Blood samples were collected, centrifuged (3000× *g*, 10 min), and serum was stored at −80 °C.

Testes were excised, weighed, and processed according to downstream applications. Samples for biochemical and molecular analyses were homogenized in Tris-HCl buffer (pH 7.4), centrifuged (10,000× *g*, 15 min, 4 °C), and supernatants were stored at −80 °C. Tissues for histology and immunohistochemistry were fixed in 10% neutral buffered formalin.

Within each experimental group, seven animals were allocated to biochemical and molecular analyses, and three to histopathological and ultrastructural examinations.

### 2.7. Assessment of Sperm Quality

Sperm parameters were evaluated from cauda epididymis samples as previously described [33]. Sperm count was determined using an Improved Neubauer hemocytometer (Chongqing New World Trading Co., Chongqing, China), motility was assessed microscopically at 37 °C, and viability and morphology were evaluated using Eosin–Nigrosin staining. At least 200 sperm per sample were analyzed. All sperm analyses were performed on coded samples by an investigator blinded to treatment allocation.

### 2.8. Assessment of Serum Hormonal Parameters

Serum testosterone, FSH, and LH levels were quantified using validated rat-specific ELISA kits according to manufacturers’ instructions. Samples were analyzed in duplicate, and concentrations were calculated from standard curves. Kit details are provided in Supplementary Table S1.

### 2.9. Assessment of Oxidative Stress and Antioxidant Markers

Testicular oxidative stress and antioxidant indices (MDA, NO, SOD, CAT, GSH) were measured using validated colorimetric assays as described previously [34]. DNA oxidative damage (8-OHdG) and antioxidant signaling markers (NRF2, HO-1) were quantified using ELISA kits [35,36]. All results were normalized to total protein content. Kit details are provided in Supplementary Table S1.

### 2.10. Evaluation of Inflammatory Mediators

Testicular levels of NF-κB, TNF-α, IL-6, and IL-1β were determined using ELISA kits following standard protocols [37]. Measurements were performed in triplicate and normalized to protein concentration.

### 2.11. Evaluation of Apoptotic Markers

Bax, Bcl-2, and caspase-3 levels were quantified using commercial ELISA kits [38]. Results were calculated from standard curves and expressed relative to total protein.

### 2.12. Evaluation of Ferroptosis-Associated Markers

Fe^2+^, GPX4, 4-HNE, ACSL4, and SLC7A11 levels were assessed using colorimetric and ELISA-based methods [39]. Lipid peroxidation was further evaluated using C11-BODIPY fluorescence [40]. Data were normalized to protein content. Kit details are listed in Supplementary Table S1.

### 2.13. RNA Extraction and RT-qPCR

Total RNA was isolated from testicular tissue using a silica-membrane-based RNA extraction kit (QIAamp RNeasy Mini Kit, Qiagen, Hilden, Germany), following the manufacturer’s instructions. On-column DNase I treatment was applied to remove residual genomic DNA. RNA concentration and purity were verified. Primers were designed using Primer-BLAST and Primer3 and synthesized by a commercial provider (Metabion, Planegg, Germany). Primer sequences, accession numbers, and amplicon lengths are listed in Table 1. In silico validation was performed to confirm specificity, and primer efficiency was determined experimentally before use. RT-qPCR was carried out using a one-step SYBR Green kit (HERA, Willowfort, Birmingham, UK) in a final reaction volume of 20 µL. Each reaction contained 10 µL of 2× SYBR Green Master Mix, 1 µL of RT enzyme mix (20×), 1 µL of each forward and reverse primer, 3 µL of nuclease-free water, and 5 µL of RNA template. Amplification was performed on a StepOne™ Real-Time PCR System (Applied Biosystems, Foster City, CA, USA) with optimized cycling conditions. Melting curve analysis was used to verify amplification specificity. The threshold cycle (Ct), also known as the quantitative cycle (Cq), was obtained using StepOne™ software v2.3. (Applied Biosystems).

### 2.14. Reference Gene Validation and Primer Efficiency

*β-Actin* was selected as the reference gene after preliminary experiments confirmed stable expression across all experimental groups, with minimal variation in Ct values and comparable expression levels to the target genes. Primer efficiency for each target and the reference gene was determined from standard curves generated using serial 10-fold dilutions of pooled testicular cDNA. Amplification efficiency (E) was calculated from the slope of the standard curve using *E* = (10^−1/slope^ − 1) × 100%, and primer pairs with efficiencies between 90 and 110% and correlation coefficients *R^2^* > 0.98 were used for expression analysis. Relative mRNA expression was calculated using the method 2^−ΔΔCt^ [41].

### 2.15. Histological Evaluation

Formalin-fixed testicular tissues were paraffin-embedded, sectioned, and stained with H&E. Histological evaluation was performed under light microscopy at 400× magnification by an experienced pathologist who was blinded to group allocation to minimize observer bias. Testicular architecture was evaluated for seminiferous tubule integrity, germ cell organization, interstitial tissue changes, and evidence of degeneration or necrosis.

### 2.16. Immunohistochemical Assessment of GPX4 and ACSL4

Paraffin-embedded testicular sections were deparaffinized in xylene and rehydrated through a graded ethanol series to distilled water. Antigen retrieval was performed in 0.01 M citrate buffer (pH 6.0) using microwave heating for 10 min, followed by cooling to room temperature. Endogenous peroxidase activity was quenched with 3% hydrogen peroxide in methanol for 10 min, and sections were rinsed in PBS.

Non-specific binding was blocked with 5% bovine serum albumin (BSA) for 30 min at room temperature. Sections were incubated overnight at 4 °C with primary antibodies against GPX4 and ACSL4 at a 1:200 dilution (Supplementary Table S1). Negative control sections were processed in parallel under identical conditions, except that the primary antibody was omitted and replaced with antibody diluent to verify staining specificity. After PBS washes, sections were incubated with a biotinylated secondary antibody for 30 min, followed by streptavidin-HRP for 20 min. Immunoreactivity was visualized using 3,3′-diaminobenzidine (DAB) as chromogen, and nuclei were counterstained with hematoxylin. Slides were mounted with DPX and examined under light microscopy at 400× magnification.

For semi-quantitative analysis, five non-overlapping fields per sample were captured digitally, and staining intensity/area was quantified using ImageJ software v. 1.54 (National Institutes of Health, Bethesda, MD, USA). The observer performing image analysis was blinded to treatment groups.

### 2.17. Statistical Analysis

Data were initially entered and checked for accuracy in Microsoft Excel and then analyzed using SAS (version 9.4; SAS Institute, Cary, NC, USA) and GraphPad Prism (version 9.0; GraphPad Software, San Diego, CA, USA). The required sample size was determined a priori using G*Power software (version 3.1.9.7), based on a one-way ANOVA with six experimental groups, an effect size of 0.40, α = 0.05, and 80% power, which indicated a minimum total sample size of 54 animals; therefore, 60 animals (10 per group) were included to ensure adequate statistical power. Normality of the distribution was assessed using the Shapiro–Wilk test, and homogeneity of variances was examined using Levene’s test before conducting parametric analyses. As the assumptions of normality and homogeneity of variance were satisfied, parametric statistical tests were considered appropriate.

Group comparisons were performed using one-way analysis of variance (ANOVA; PROC ANOVA in SAS). When a significant main effect was detected, Tukey’s honestly significant difference (HSD) post hoc test was applied for pairwise comparisons to control for multiple testing. The experimental unit was the individual animal for all analyses. Data are presented as mean ± standard error of the mean (SEM). A two-tailed *p* < 0.05 was considered statistically significant. All statistical analyses were prespecified before data evaluation. No data transformation was performed, and no observations were excluded from the statistical analyses.

## 3. Results

### 3.1. Physicochemical Characterization of HES-PLGA-NPs

**Figure 1:**
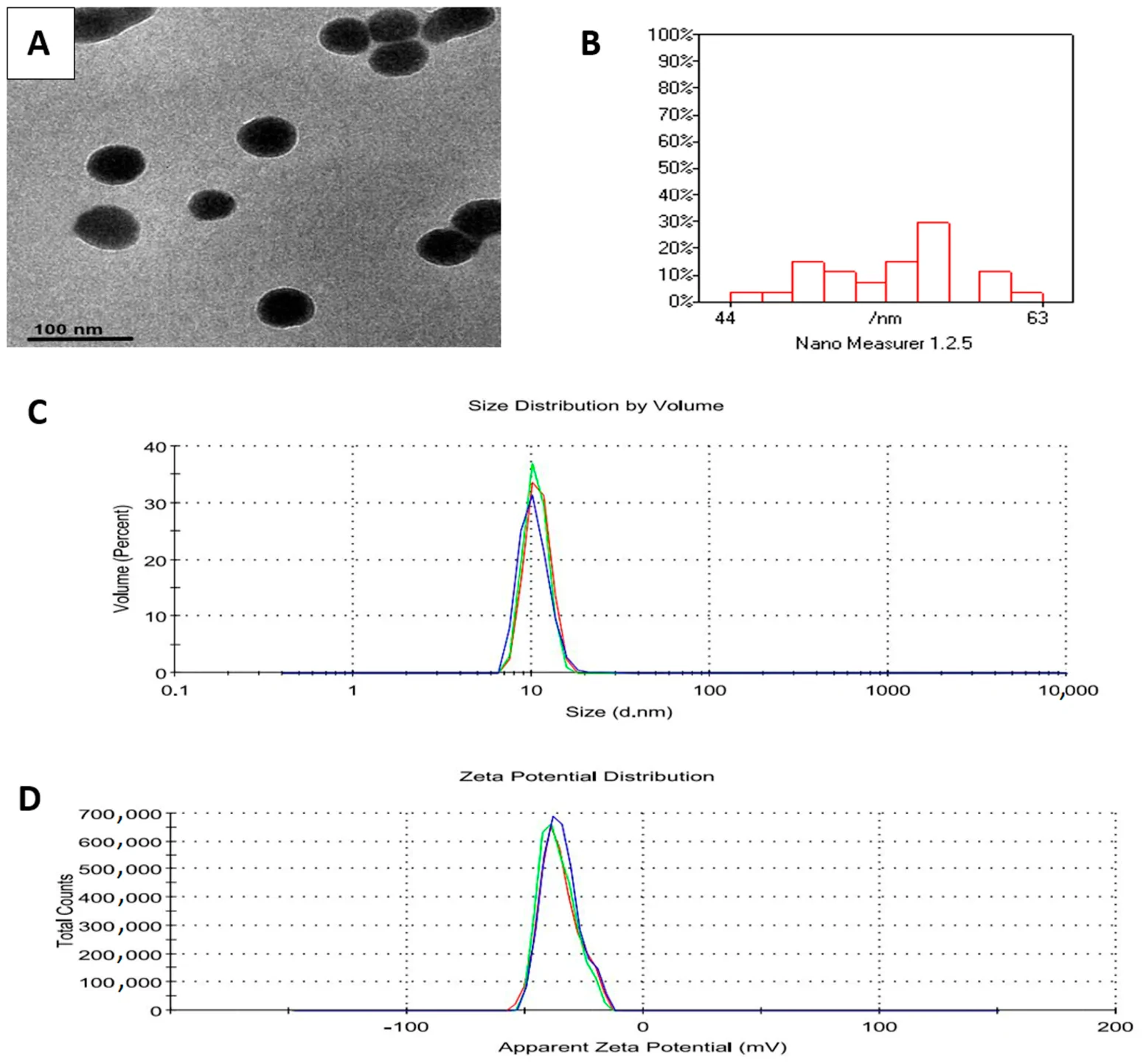
Physicochemical characterization of hesperetin-loaded PLGA nanoparticles (HES-PLGA-NPs). (**A**) Transmission electron microscopy (TEM) image showing spherical nanoparticles. (**B**) Particle-size distribution histogram showing particle diameters ranging from 44 to 63 nm. (**C**) Volume-based particle size distribution; the red, green, and blue curves represent three independent replicate measurements. (**D**) Zeta potential distribution; the red, green, and blue curves represent three independent replicate measurements.

As shown in Figure 1A, HES-PLGA-NPs exhibited a predominantly spherical morphology with discrete, non-aggregated particles. The particle size distribution ranged from 44 to 63 nm, indicating a relatively narrow size distribution (Figure 1B). Dynamic light scattering revealed an average hydrodynamic diameter of 49 nm and a low polydispersity index (PDI = 0.201), confirming the formation of a homogeneous nanosuspension (Figure 1C). The zeta potential was −39.13 mV (Figure 1D), reflecting strong electrostatic repulsion and suggesting good colloidal stability. The formulation also showed a high entrapment efficiency (EE) of 88.17%, indicating efficient incorporation of HES within the PLGA matrix.

### 3.2. In Vitro Release Profile of Hesperetin from HES-PLGA-NPs

**Figure 2:**
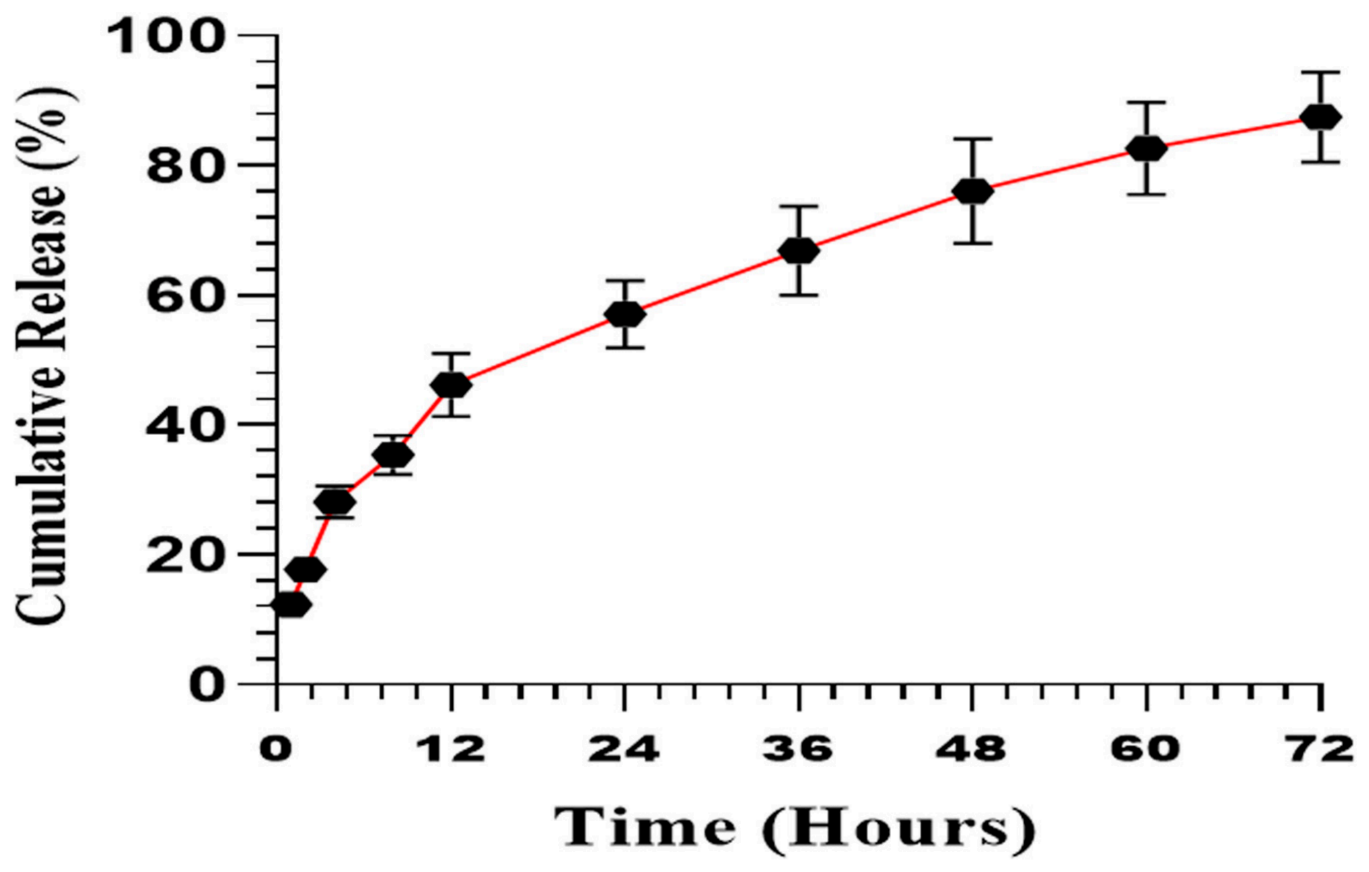
In vitro release profile of HES from HES-PLGA-NPs in PBS (pH 7.4) at 37 °C using the dialysis membrane diffusion method. Data are expressed as mean ± SD (n = 3).

The in vitro release profile of HES from HES-PLGA-NPs is presented in Figure 2. An initial burst release was observed during the first 8 h, with approximately 35.4% of the encapsulated drug released into the medium. This phase was followed by a slower, sustained release pattern, with cumulative release reaching 57.1% at 24 h and gradually increasing to 87.5% by 72 h. The biphasic release behavior is consistent with diffusion of surface-associated HES, followed by controlled release from the PLGA core, and supports the formulation’s suitability for sustained delivery.

### 3.3. Fourier-Transform Infrared Spectroscopy (FTIR) Characterization of HES-PLGA-NPs

**Figure 3:**
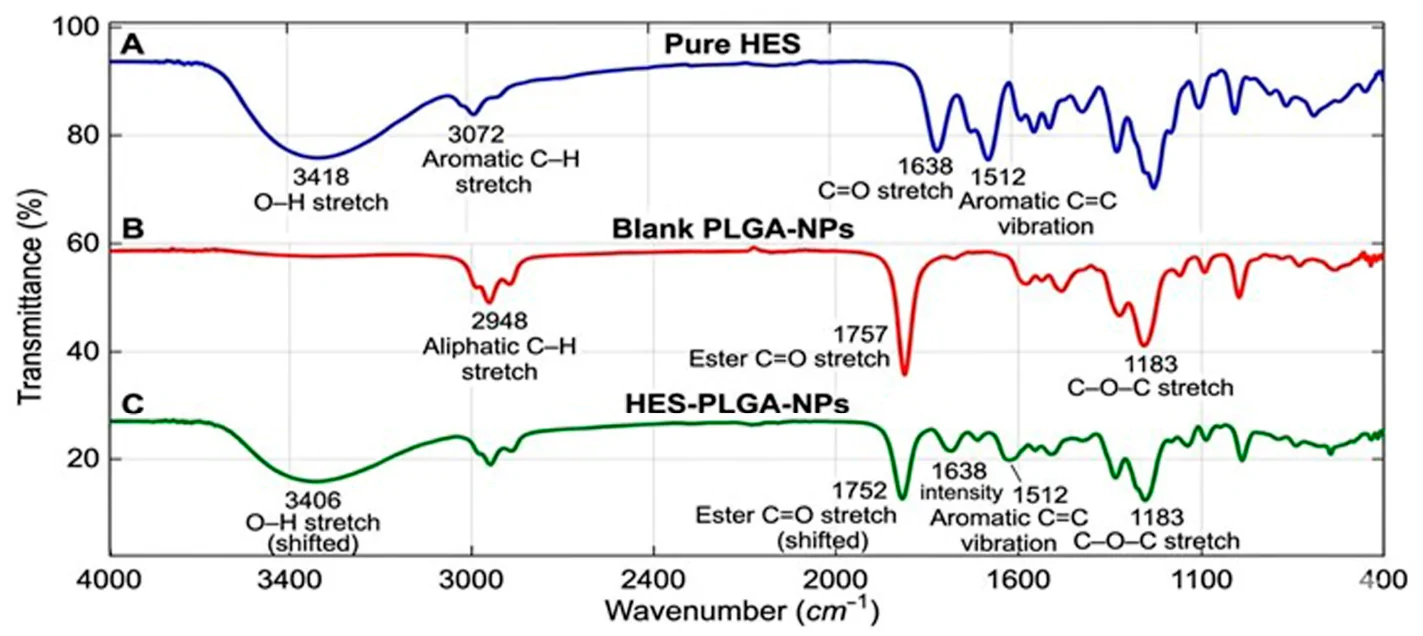
FTIR spectra of pure HES, blank PLGA nanoparticles, and HES-PLGA-NPs, confirming successful HES loading into PLGA with slight band shifts and reduced HES peak intensity.

FTIR spectra of pure HES, blank PLGA nanoparticles, and HES-PLGA-NPs are shown in Figure 3. Pure HES displayed a broad O–H stretching band at 3418 cm^−1^, along with characteristic bands at 3072 cm^−1^ (aromatic C–H), 1638 cm^−1^ (C=O stretching), and 1512 cm^−1^ (aromatic C=C). Blank PLGA nanoparticles showed the typical ester carbonyl peak at 1757 cm^−1^ and bands at 2948 and 1183 cm^−1^ corresponding to aliphatic C–H and C–O–C stretching, respectively. In HES-PLGA-NPs, the main bands of both HES and PLGA were preserved but exhibited slight shifts (O–H from 3418 to 3406 cm^−1^, carbonyl from 1757 to 1752 cm^−1^), together with a noticeable reduction in the intensity of several HES-related peaks. No new absorption bands were detected. These findings indicate successful entrapment of HES within the PLGA matrix, with likely weak intermolecular interactions, such as hydrogen bonding, but no evidence of new covalent bond formation or structural modification of the drug.

### 3.4. Effect of HES-PLGA-NPs on Reproductive Hormones

**Figure 4:**
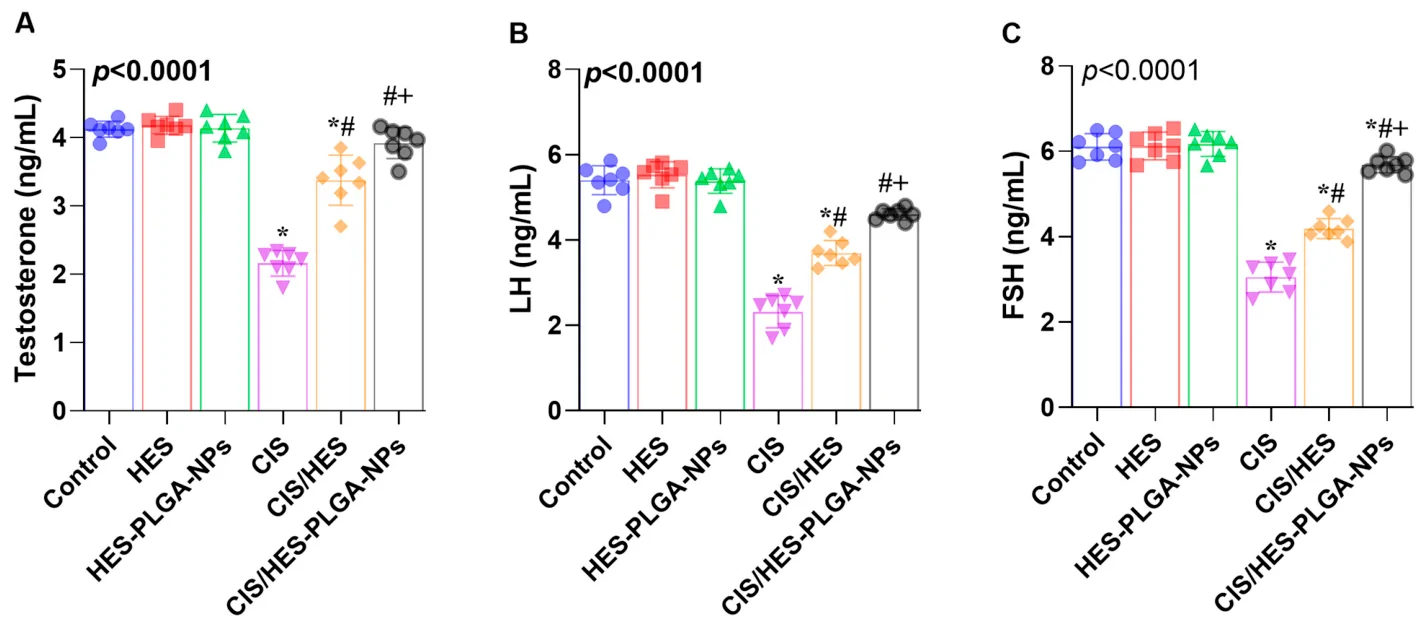
Effects of hesperetin (HES) and hesperetin-loaded poly (lactic-co-glycolic acid) nanoparticles (HES-PLGA-NPs) on serum reproductive hormones in cisplatin-induced testicular toxicity. (**A**) Testosterone, (**B**) Luteinizing hormone (LH), and (**C**) Follicle-stimulating hormone (FSH) in control, HES, HES-PLGA-NPs, cisplatin (CIS), CIS/HES, and CIS/HES-PLGA-NPs groups. Data are expressed as mean ± SEM (n = 7 rats per group). Statistical analysis was performed using one-way ANOVA followed by Tukey’s HSD post hoc test (overall *p* < 0.000 for each hormone). Different superscripts denote significant differences (*p* < 0.05) relative to the control (*), CIS (#), and free HES (+) groups.

Cisplatin (CIS) administration markedly disrupted the reproductive hormonal profile, as evidenced by significant reductions in serum testosterone, LH, and FSH levels compared with the control group (all adjusted *p* < 0.0001; Figure 4A–C). Testosterone decreased by 44.5%, LH by 54.2%, and FSH by 45.5% relative to the control group. Co-treatment with free HES significantly ameliorated these hormonal disturbances, increasing testosterone (1.54-fold), LH (1.48-fold), and FSH (1.28-fold) compared with the CIS group (all *p* < 0.0001). HES-PLGA-NPs produced a significantly greater restorative effect than the free HES, increasing testosterone (1.78-fold; *p* < 0.0001 vs. CIS + HES), (1.73-fold; *p* < 0.0001), and FSH (1.36-fold; *p* < 0.0001). Importantly, serum testosterone in the CIS + HES-PLGA-NPs group was restored to a level comparable with the control group (*p* = 0.990), whereas the CIS + HES group remained significantly lower than the control (*p* < 0.0001). Likewise, FSH concentrations were substantially closer to the control values following HES-PLGA-NP treatment (mean difference = 0.332, *p* = 0.029) than after crude HES treatment (mean difference = 1.860, adjusted *p* < 0.0001), while LH levels also showed greater recovery with HES-PLGA-NPs than with free HES (*p* < 0.0001 for CIS + HES vs. CIS + HES-PLGA-NPs). However, they remained significantly below control values (*p* < 0.0001).

### 3.5. Effect of HES-PLGA-NPs on Testicular Weight and Sperm Quality

As summarized in Table 2, CIS significantly reduced testicular weight by 28.6% compared with the control group. Co-treatment with HES and HES-PLGA-NPs increased testicular weight by 1.18- and 1.32-fold, respectively, versus CIS, with the greatest improvement observed in the CIS + HES-PLGA-NPs group.

CIS also markedly impaired semen quality, reducing sperm concentration, progressive sperm motility, and sperm viability by 42.3%, 46.8%, and 38.9%, respectively, while increasing the percentage of morphologically abnormal spermatozoa by 2.89-fold compared with the control group. HES improved sperm concentration, progressive motility, and viability by 1.36-, 1.40-, and 1.41-fold, respectively, and reduced morphological abnormalities by 18.5% versus CIS. HES-PLGA-NPs produced greater recovery, increasing sperm concentration, progressive motility, and viability by 1.55-, 1.70-, and 1.54-fold, respectively, while reducing the percentage of morphologically abnormal spermatozoa by 33.6% compared with CIS. These findings indicate that HES-PLGA-NPs provided stronger protection of testicular structure and sperm quality parameters than crude HES.

### 3.6. Effect of HES-PLGA-NPs on Oxidative Stress, Nrf2/Ho-1 Signaling, and Antioxidant Defenses

**Figure 5:**
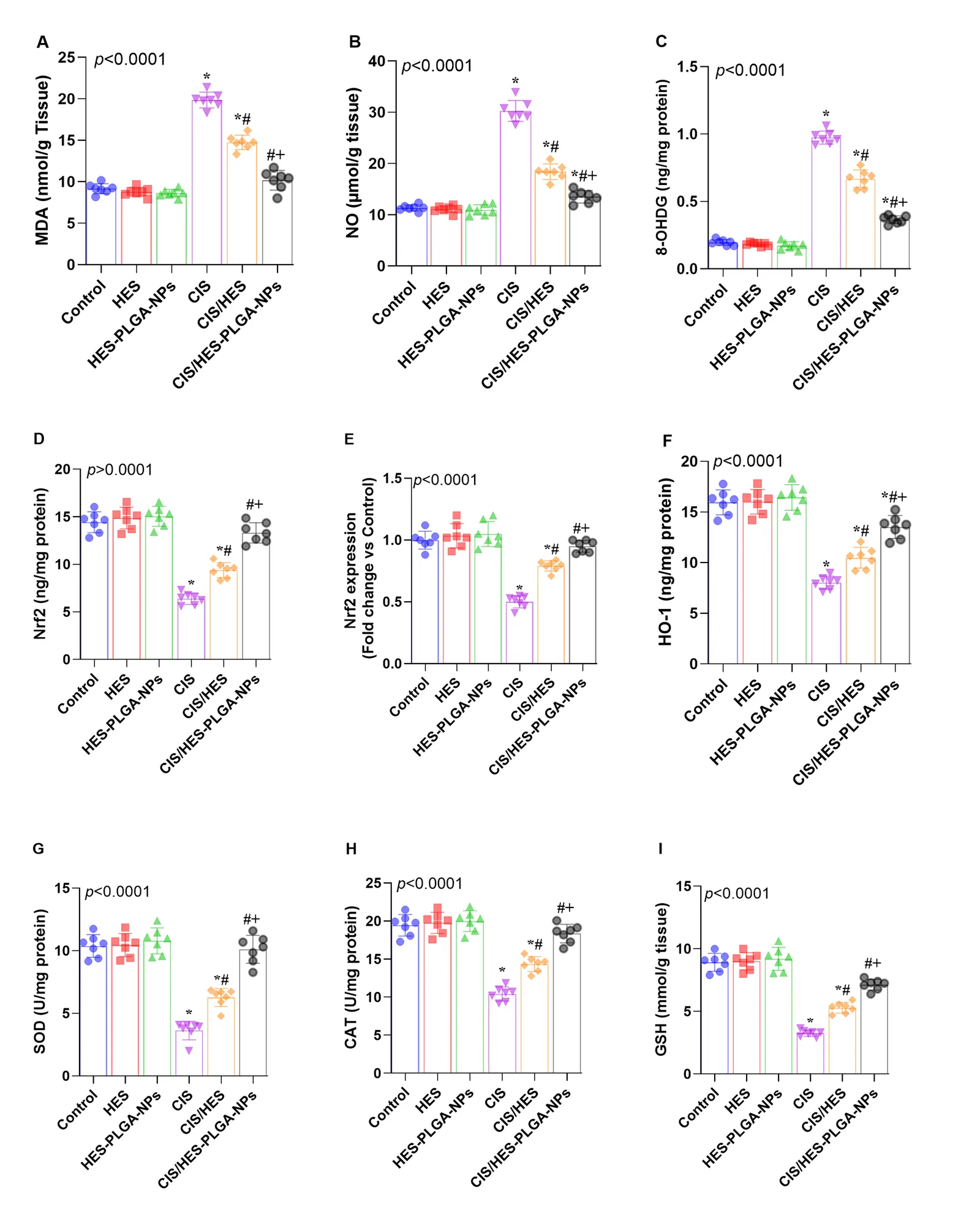
Effects of hesperetin (HES) and hesperetin-loaded poly (lactic-co-glycolic acid) nanoparticles (HES-PLGA-NPs) on oxidative stress biomarkers, NRF2/HO-1 signaling, and antioxidant defenses in testicular tissue of cisplatin (CIS)-treated rats. (**A**) Malondialdehyde (MDA), (**B**) Nitric oxide (NO), (**C**) 8-hydroxy-2′-deoxyguanosine (8-OHdG), (**D**) Nuclear factor erythroid 2-related factor 2 (NRF2) protein, (**E**) NRF2 mRNA, (**F**) Heme oxygenase-1 (HO-1), (**G**) Superoxide dismutase (SOD), (**H**) Catalase (CAT), and (**I**) Reduced glutathione (GSH). Data are expressed as mean ± SEM (n = 7 rats per group). Statistical analysis was performed using one-way ANOVA followed by Tukey’s HSD post hoc test (overall *p* < 0.000 for each marker). Different superscripts denote significant differences (*p* < 0.05) relative to the control (*), CIS (#), and free HES (+) groups.

CIS treatment markedly increased testicular MDA, NO, and 8-OHdG levels compared with control by 2.16-, 2.58-, and 4.78-fold, respectively (all adjusted *p* < 0.0001; Figure 5A–C), in line with severe oxidative stress and oxidative DNA damage. Co-treatment with free HES significantly reduced MDA, NO, and 8-OHdG compared with CIS alone by 25.7%, 37.0%, and 28.7%, respectively (all *p* < 0.0001). However, HES-PLGA-NPs produced a stronger reduction in these markers by 49.0%, 52.8%, and 59.6%, respectively, versus CIS (all adjusted *p* < 0.0001). Direct comparison showed that HES-PLGA-NPs were associated with significantly lower MDA, NO, and 8-OHdG levels in the CIS + HES-PLGA-NPs group than in the CIS + HES group (adjusted *p* < 0.0001, *p* = 0.0003, and *p* < 0.0001, respectively). Notably, MDA and NO levels in the CIS + HES-PLGA-NPs group were not significantly different from those in the control group (*p* = 0.447 and 0.112, respectively).

CIS also significantly suppressed NRF2 protein, NRF2 mRNA, and HO-1 protein levels compared with the control by 55.6%, 47.3%, and 49.7%, respectively (all *p* < 0.0001; Figure 5D–F). Free HES significantly restored NRF2 protein, NRF2 mRNA, and HO-1 levels by 1.47-, 1.53-, and 1.31-fold, respectively, compared with CIS (all adjusted *p* < 0.001). HES-PLGA-NPs showed greater restoration, increasing these parameters by 2.09-, 1.82-, and 1.68-fold, respectively, versus CIS (all *p* < 0.0001). The nanoformulation was significantly more effective than crude HES in restoring NRF2 protein, NRF2 mRNA, and HO-1 levels (all *p* < 0.0001). NRF2 protein and NRF2 mRNA levels in the CIS + HES-PLGA-NPs group were not significantly different from control (*p* = 0.254 and *p* = 0.081, respectively).

Consistently, CIS significantly reduced SOD, CAT, and GSH compared with control by 65.5%, 46.3%, and 63.3%, respectively (all *p* < 0.0001; Figure 5G–I). Free HES significantly increased SOD, CAT, and GSH by 1.73-, 1.37-, and 1.62-fold, respectively, versus CIS (all *p* < 0.0001). HES-PLGA-NPs produced a stronger recovery, increasing SOD, CAT, and GSH by 2.86-, 1.76-, and 2.18-fold, respectively, compared with CIS (all *p* < 0.0001). Direct comparison showed significantly higher SOD, CAT, and GSH levels in the CIS + HES-PLGA-NPs group than in the CIS + HES group (all *p* < 0.0001), confirming the superior antioxidant-restorative efficacy of the nanoformulation. SOD and CAT levels in the CIS + HES-PLGA-NPs group were comparable to the control (*p* = 0.9993 and *p* = 0.3807, respectively).

### 3.7. Effect of HES-PLGA-NPs on NF-κB Signaling and Inflammatory Mediators

**Figure 6:**
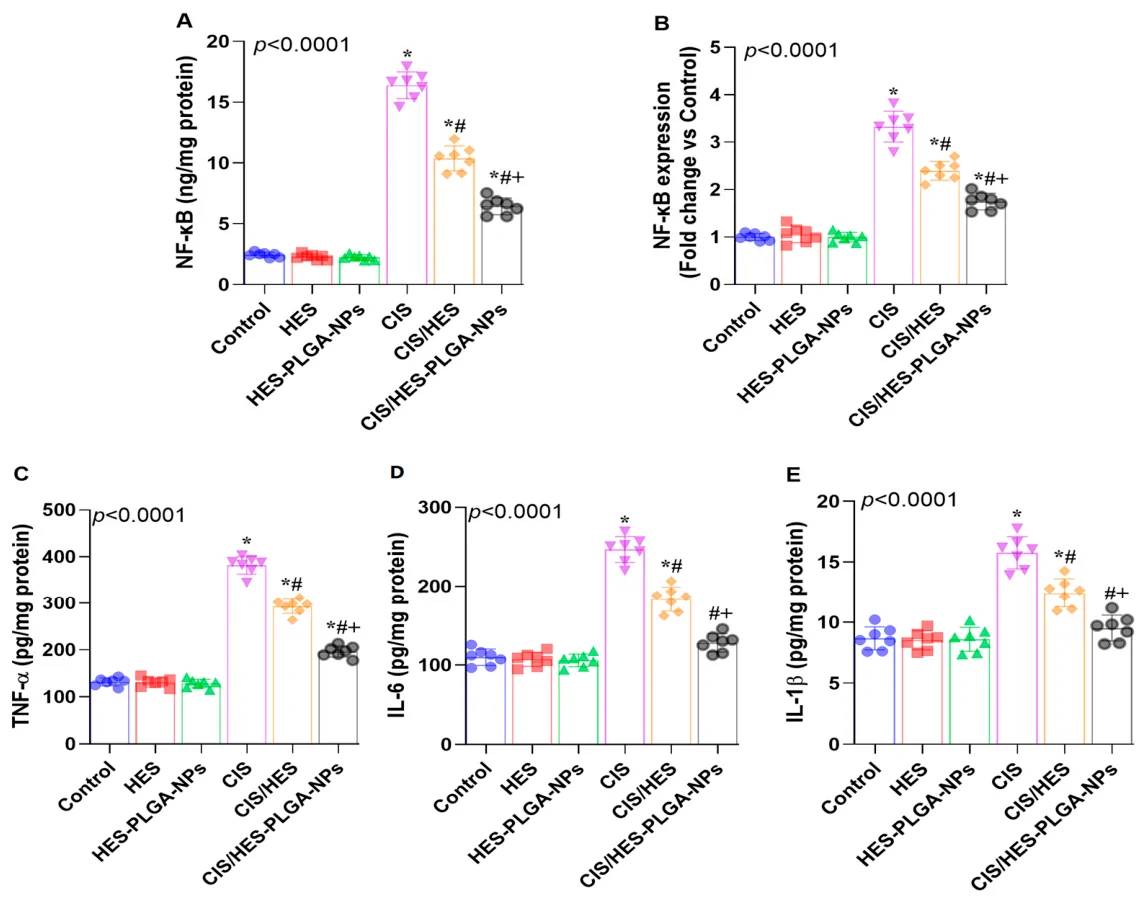
Effects of hesperetin (HES) and hesperetin-loaded poly (lactic-co-glycolic acid) nanoparticles (HES-PLGA-NPs) on nuclear factor kappa B (NF-κB) signaling and inflammatory mediators in testicular tissue of cisplatin (CIS)-treated rats. (**A**) NF-κB protein, (**B**) NF-κB mRNA, (**C**) Tumor necrosis factor-alpha (TNF-α), (**D**) Interleukin-6 (IL-6), and (**E**) Interleukin-1 beta (IL-1β). Data are expressed as mean ± SEM (n = 7 rats per group). Statistical analysis was performed using one-way ANOVA followed by Tukey’s HSD post hoc test (overall *p* < 0.000). Different superscripts denote significant differences (*p* < 0.05) versus control (*), CIS (#), and free HES (+).

CIS exposure was associated with marked activation of testicular NF-κB signaling, as shown by significant increases in NF-κB protein and NF-κB mRNA expression by 6.69- and 3.42-fold, respectively, compared with control (both adjusted *p* < 0.0001; Figure 6A,B). Co-treatment with free HES significantly reduced NF-κB protein and mRNA expression by 36.5% and 29.2%, respectively, versus CIS (both adjusted *p* < 0.0001). HES-PLGA-NPs produced a stronger inhibitory effect, reducing NF-κB protein and mRNA expression by 60.6% and 47.8%, respectively, compared with CIS (both adjusted *p* < 0.0001). A direct comparison showed that HES-PLGA-NPs were significantly associated with downregulation of NF-κB protein and gene expression compared with free HES (both adjusted *p* < 0.0001).

In parallel, CIS markedly elevated testicular TNF-α, IL-6, and IL-1β levels by 2.89-, 2.24-, and 1.80-fold, respectively, compared with control (all adjusted *p* < 0.0001; Figure 6C–E). Free HES significantly reduced TNF-α, IL-6, and IL-1β by 23.3%, 25.5%, and 21.0%, respectively, versus CIS (all adjusted *p* < 0.0001). HES-PLGA-NPs exerted a significantly greater anti-inflammatory effect, reducing these cytokines by 48.1%, 48.0%, and 39.5%, respectively, compared with CIS (all adjusted *p* < 0.0001). The nanoformulation was also significantly superior to crude HES in lowering TNF-α, IL-6, and IL-1β levels (adjusted *p*-values < 0.0001, < 0.0001, and 0.0001, respectively). Notably, IL-1β levels in the CIS + HES-PLGA-NPs group were not significantly different from those in the control group (adjusted *p* = 0.692), indicating near-normalization.

### 3.8. Effect of HES-PLGA-NPs on Apoptotic Markers

**Figure 7:**
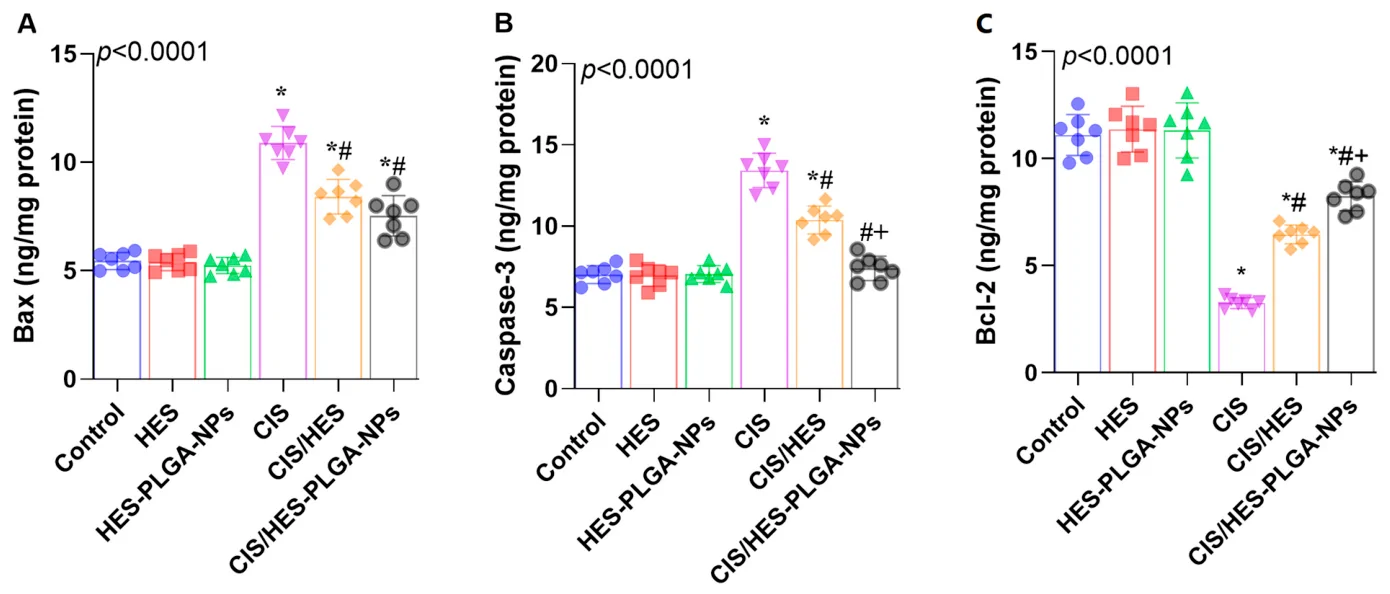
Effects of hesperetin (HES) and hesperetin-loaded poly (lactic-co-glycolic acid) nanoparticles (HES-PLGA-NPs) on apoptotic markers in testicular tissue of cisplatin (CIS)-treated rats. (**A**) Bcl-2-associated X protein (Bax). (**B**) Cysteinyl aspartate-specific protease-3 (caspase-3), and (**C**) B-cell lymphoma-2 (Bcl-2). Data are expressed as mean ± SEM (n = 7 rats per group). Statistical analysis was performed using one-way ANOVA followed by Tukey’s HSD post hoc test (overall *p* < 0.000). Different superscripts denote significant differences (*p* < 0.05) versus control (*), CIS (#), and free HES (+).

CIS exposure significantly increased testicular Bax and caspase-3 levels by 1.98- and 1.91-fold, respectively, compared with control (both *p* < 0.0001; Figure 7A,B), indicating enhanced pro-apoptotic signaling. Co-treatment with free HES significantly reduced Bax and caspase-3 by 21.7% and 22.6%, respectively, versus CIS (both *p* < 0.0001). HES-PLGA-NPs produced a stronger reduction, decreasing Bax and caspase-3 by 29.6% and 44.8%, respectively, compared with CIS (both *p* < 0.0001). However, the reduction in Bax did not differ significantly between CIS + HES and CIS + HES-PLGA-NPs (*p* = 0.181), whereas caspase-3 was significantly lower in the CIS + HES-PLGA-NPs group than in the CIS + HES group (*p* < 0.0001). Notably, caspase-3 levels in the CIS + HES-PLGA-NPs group were not significantly different from those in the control group (*p* = 0.897).

Conversely, CIS significantly decreased the anti-apoptotic protein Bcl-2 by 70.7% compared with the control (*p* < 0.0001; Figure 7C). Free HES significantly increased Bcl-2 by 1.98-fold versus CIS (*p* < 0.0001), while HES-PLGA-NPs produced a greater increase of 2.53-fold compared with CIS (*p* < 0.0001). Direct comparison showed significantly higher Bcl-2 levels in the CIS + HES-PLGA-NPs group than in the CIS + HES group (*p* = 0.001), supporting the nanoformulation’s association with a favorable anti-apoptotic effect.

### 3.9. Effect of HES-PLGA-NPs on Ferroptosis-Associated Markers

**Figure 8:**
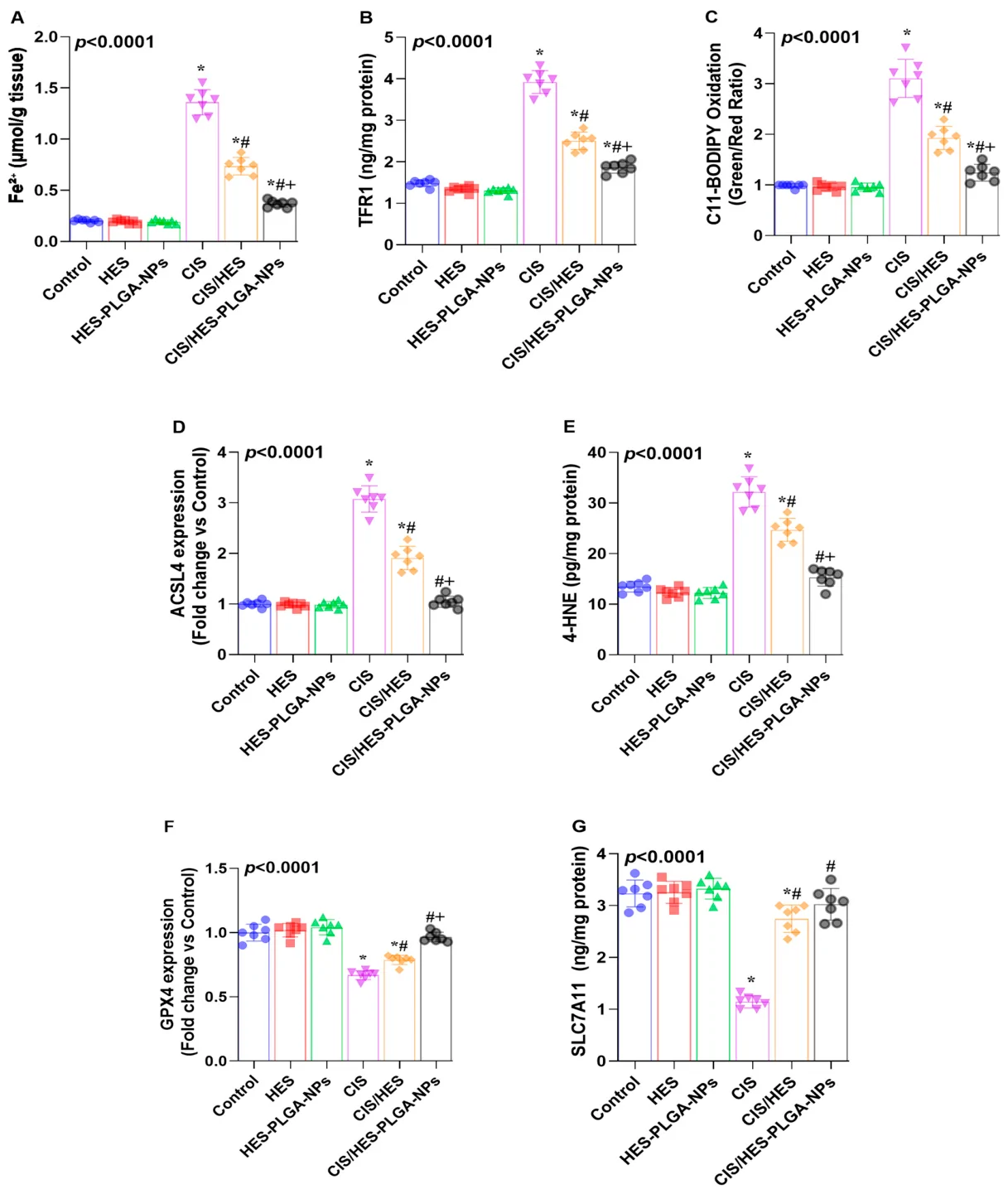
Effects of hesperetin (HES) and hesperetin-loaded poly (lactic-co-glycolic acid) nanoparticles (HES-PLGA-NPs) on ferroptosis-associated markers in testicular tissue of cisplatin (CIS)-treated rats. (**A**) Ferrous iron (Fe^2+^), (**B**) Transferrin receptor 1 (TFR1), (**C**) Lipid-derived reactive oxygen species (ROS), (**D**) Acyl-CoA synthetase long-chain family member 4 (ACSL4) mRNA, (**E**) 4-hydroxynonenal (4-HNE), (**F**) Glutathione peroxidase 4 (GPX4) mRNA, and (**G**) Solute carrier family 7 member 11 (SLC7A11). Data are expressed as mean ± SEM (n = 7 rats per group). Statistical analysis was performed using one-way ANOVA followed by Tukey’s HSD post hoc test (overall *p* < 0.000). Different superscripts denote significant differences (*p* < 0.05) versus control (*), CIS (#), and free HES (+).

CIS treatment significantly increased testicular Fe content, TFR1 levels, lipid ROS, ACSL4 mRNA expression, and 4-HNE concentrations by 6.72-, 2.65-, 3.18-, 3.08-, and 2.39-fold, respectively, compared with control (all *p* < 0.0001; Figure 8A–E), indicating ferroptosis-associated iron overload and lipid peroxidation. Free HES significantly reduced Fe, TFR1, lipid ROS, ACSL4, and 4-HNE by 45.9%, 36.1%, 37.9%, 36.5%, and 23.3%, respectively, versus CIS (all *p* < 0.0001). HES-PLGA-NPs produced greater reductions of 72.9%, 52.5%, 59.9%, 65.7%, and 52.9%, respectively, compared with CIS (all *p* < 0.0001). Direct comparison confirmed that HES-PLGA-NPs were significantly more effective than crude HES in lowering Fe, TFR1, lipid ROS, ACSL4, and 4-HNE levels (all *p* < 0.0001). Notably, lipid ROS, ACSL4 mRNA expression, and 4-HNE concentrations in the CIS + HES-PLGA-NPs group were not significantly different from control (*p* = 0.152, *p* = 0.987, and *p* = 0.485, respectively).

Conversely, CIS significantly suppressed GPX4 mRNA expression and SLC7A11 levels by 32.0% and 64.5%, respectively, compared with control (both *p* < 0.0001; Figure 8F,G). Free HES significantly increased GPX4 and SLC7A11 by 1.18- and 2.33-fold, respectively, versus CIS (both *p* < 0.0001). HES-PLGA-NPs showed stronger restoration, increasing GPX4 and SLC7A11 by 1.43- and 2.64-fold, respectively, compared with CIS (both *p* < 0.0001). The nanoformulation was significantly superior to crude HES in restoring GPX4 and SLC7A11 expression (*p* < 0.0001 and *p* = 0.029, respectively). Importantly, GPX4 and SLC7A11 levels in the CIS + HES-PLGA-NPs group were comparable to those in the control group (*p* = 0.478 and *p* = 0.525, respectively), supporting effective suppression of ferroptotic signaling.

### 3.10. Effect of HES-PLGA-NPs on Histopathological Alterations

**Figure 9:**
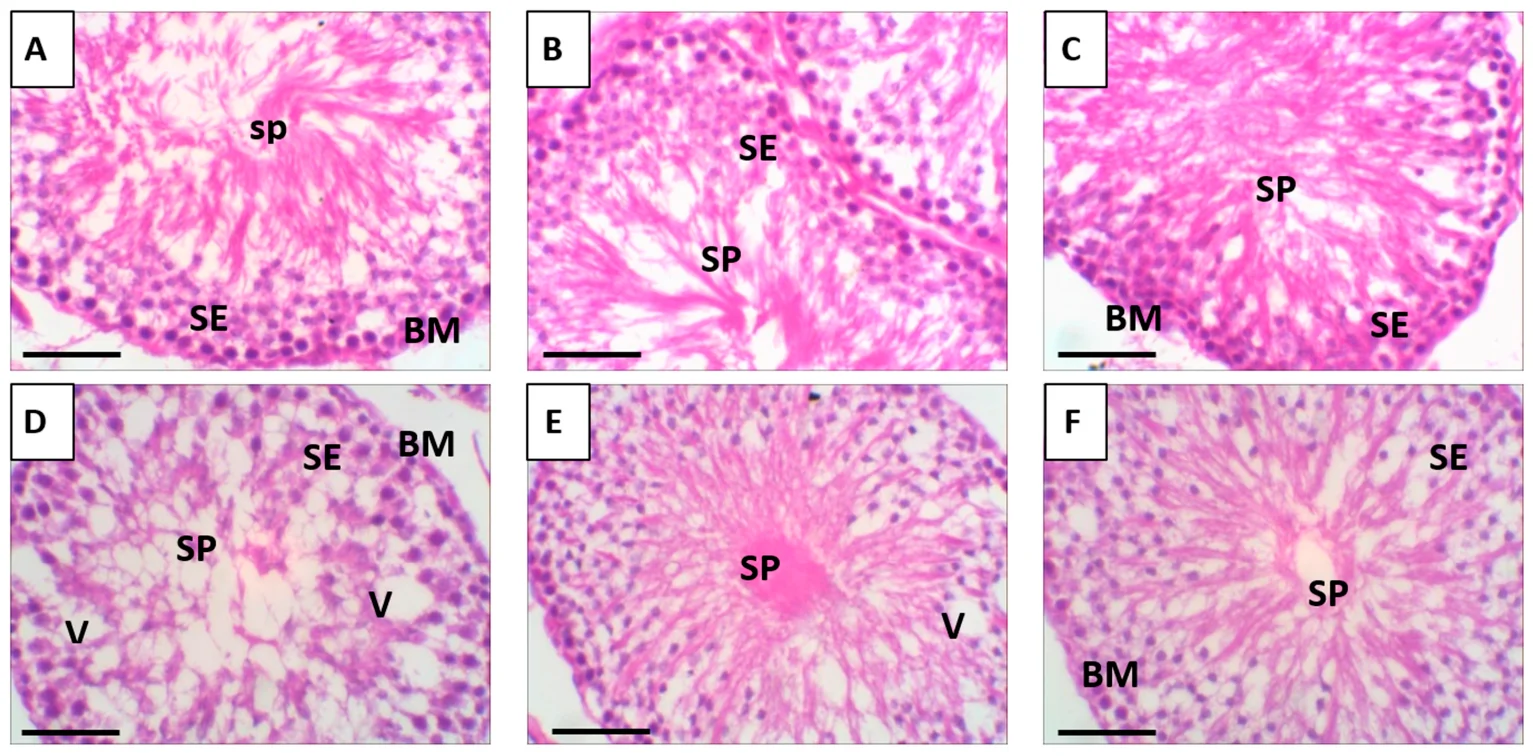
Representative H&E-stained sections of rat testes. (**A**) Control, (**B**) hesperetin (HES), (**C**) hesperetin-loaded poly (lactic-co-glycolic acid) nanoparticles (HES-PLGA-NPs), (**D**) cisplatin (CIS), (**E**) CIS + HES, and (**F**) CIS + HES-PLGA-NPs. BM, basement membrane; SP, spermatozoa; SE, seminiferous epithelium; V, cytoplasmic vacuolation. Magnification ×400; scale bar = 50 µm (shown in each panel).

Histological examination of control, HES, and HES-PLGA-NPs groups revealed normal testicular architecture with intact seminiferous tubules, well-organized germinal epithelium, and active spermatogenesis (Figure 9A–C). In contrast, CIS-treated rats showed marked disruption of seminiferous tubule structure, extensive separation and thinning of the germinal epithelium, and pronounced nuclear changes, including pyknosis and karyorrhexis, consistent with severe testicular injury (Figure 9D).

Co-treatment with HES or HES-PLGA-NPs mitigated CIS-induced damage. Testes from CIS + HES and CIS + HES-PLGA-NPs groups exhibited better preserved seminiferous tubules, improved germ cell organization, and only mild vacuolation and focal thinning of the spermatogenic epithelium (Figure 9E,F). The CIS + HES-PLGA-NPs group showed the closest resemblance to the normal histological pattern, with an almost intact germinal epithelium and ongoing spermatogenesis.

### 3.11. Effect of HES-PLGA-NPs on GPX4 Immunohistochemical Expression

**Figure 10:**
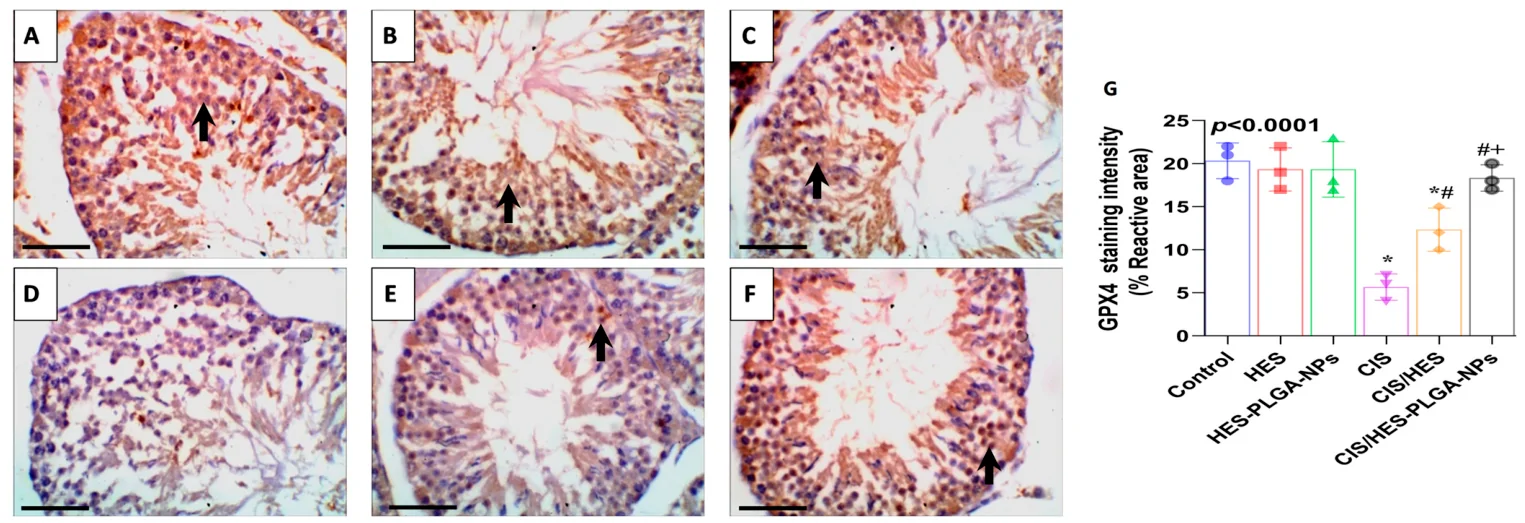
Representative photomicrographs of glutathione peroxidase 4 (GPX4) immunostaining in testicular tissue. (**A**) Control, (**B**) hesperetin (HES), (**C**) hesperetin-loaded poly (lactic-co-glycolic acid) nanoparticles (HES-PLGA-NPs), (**D**) Cisplatin (CIS), (**E**) CIS + HES, and (**F**) CIS + HES-PLGA-NPs. Magnification ×400; scale bar = 50 µm (shown in each panel). (**G**) Quantitative GPX4 immunoscore in testes of control and experimental groups. Data are expressed as mean ± SEM (n = 3 rats per group). Statistical analysis was performed using one-way ANOVA followed by Tukey’s HSD post hoc test (overall *p* < 0.000). Different superscripts denote significant differences (*p* < 0.05) versus control (*), CIS (#), and free HES (+).

GPX4 immunostaining was strongly positive throughout the germinal epithelium in control, HES, and HES-PLGA-NPs groups (Figure 10A–C). In contrast, CIS-treated testes showed marked attenuation of GPX4 immunoreactivity, with weak staining in germ cells (Figure 10D). Co-administration of HES or HES-PLGA-NPs with CIS improved GPX4 expression, with more intense and widespread staining observed in both treatment groups, particularly in the CIS + HES-PLGA-NPs group (Figure 10E,F).

Quantitative analysis (Figure 10G) demonstrated that the GPX4 immunoscore was significantly reduced by 72.1% in the CIS group compared with the control group (*p* < 0.0001). Treatment with HES significantly increased the GPX4 immunoscore by 2.18-fold relative to CIS (*p* = 0.037), while HES-PLGA-NPs produced a greater 3.24-fold increase (*p* = 0.0002). Although the GPX4 immunoscore was numerically higher in the CIS + HES-PLGA-NPs group than in the CIS + HES group (48.6% increase), this difference did not reach statistical significance (*p* = 0.067). Importantly, GPX4 immunoscores in the CIS + HES-PLGA-NPs group were not significantly different from those of the control group (*p* = 0.887), indicating near-complete restoration of GPX4 expression.

### 3.12. Effect of HES-PLGA-NPs on ACSL4 Immunohistochemical Expression

**Figure 11:**
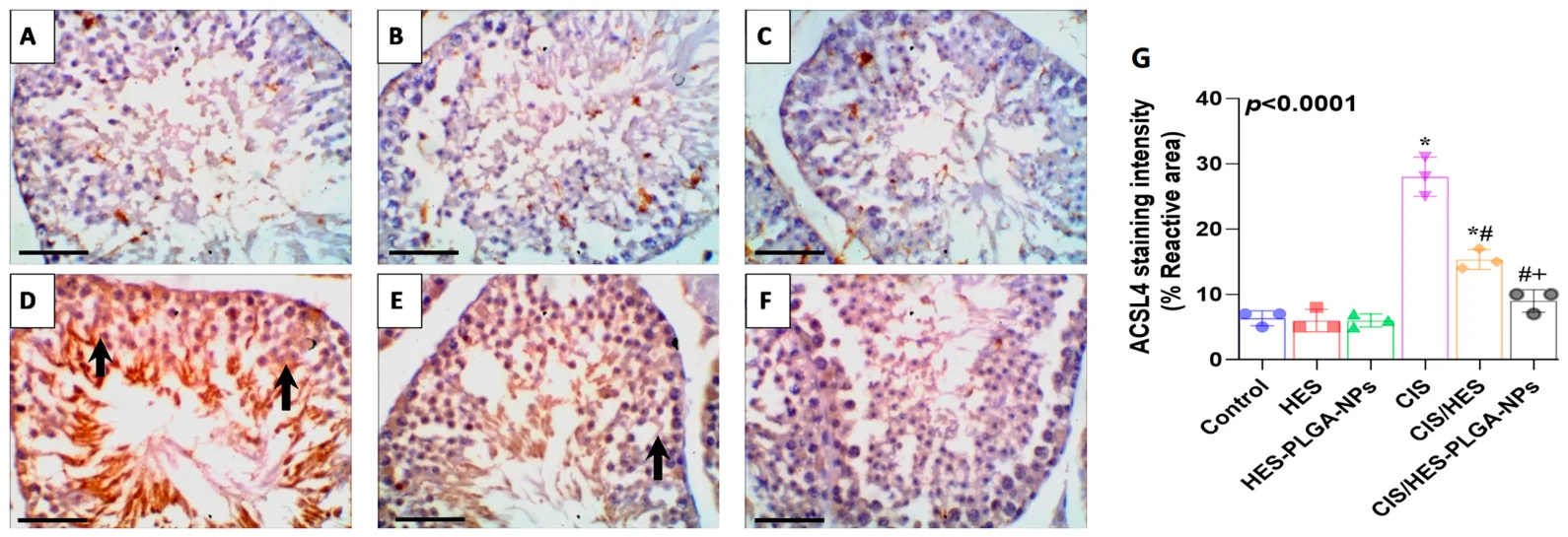
Representative photomicrographs of acyl-CoA synthetase long-chain family member 4 (ACSL4) immunostaining in testicular tissue. (**A**) Control, (**B**) hesperetin (HES), (**C**) hesperetin-loaded poly (lactic-co-glycolic acid) nanoparticles (HES-PLGA-NPs), (**D**) Cisplatin (CIS), (**E**) CIS + HES, and (**F**) CIS + HES-PLGA-NPs. Magnification ×400; scale bar = 50 µm (shown in each panel). (**G**) Quantitative ACSL4 immuno-score in testes of control and experimental groups. Data are expressed as mean ± SEM (n = 3 rats per group). Statistical analysis was performed using one-way ANOVA followed by Tukey’s HSD post hoc test (overall *p* < 0.000). Different superscripts denote significant differences (*p* < 0.05) versus control (*), CIS (#), and free HES (+).

In the control, HES, and HES-PLGA-NPs groups, ACSL4 immunostaining was absent or negligible in germ cells (Figure 11A–C). CIS treatment markedly increased ACSL4 immunoreactivity, with intense brown staining throughout the germinal epithelium, consistent with ferroptosis activation (Figure 11D). Co-treatment with HES or HES-PLGA-NPs substantially reduced ACSL4 staining intensity compared with CIS alone (Figure 11E,F). The staining pattern in the CIS + HES-PLGA-NPs group closely resembled that of the control group.

Quantitative analysis (Figure 11G) demonstrated that the ACSL4 immunoscore was significantly increased by 4.42-fold in the CIS group compared with the control group (*p* < 0.0001). Treatment with HES significantly reduced the ACSL4 immunoscore by 45.3% relative to CIS (*p* < 0.0001), whereas HES-PLGA-NPs produced a greater 67.9% reduction (*p* < 0.0001). Moreover, the ACSL4 immunoscore was significantly lower in the CIS + HES-PLGA-NPs group than in the CIS + HES group (41.3% reduction, *p* = 0.011). Importantly, ACSL4 immunoscores in the CIS + HES-PLGA-NPs group were not significantly different from those in the control group (*p* = 0.498), indicating near-complete normalization of ACSL4 expression.

## 4. Discussion

The present study demonstrates that cisplatin exerts profound testicular toxicity, reflected by reduced testicular weight, impaired sperm parameters, and marked histological injury. These changes are indicative of germ cell loss, shrinkage and disorganization of seminiferous tubules, and degeneration of the interstitial compartment. Such findings are consistent with previous reports showing that cisplatin preferentially damages rapidly proliferating spermatogenic cells through mitochondrial dysfunction, DNA adduct formation, and disruption of cell cycle regulation [1,2]. In line with these mechanisms, we observed significant declines in sperm count, progressive motility, and viability, along with increased abnormal sperm forms, indicating a global failure of spermatogenesis and epididymal maturation. Germ cells are highly vulnerable to oxidative stress owing to their rich polyunsaturated fatty acid content and relatively weak intrinsic antioxidant defenses [8,42]. Excessive ROS generation triggered by cisplatin therefore readily compromises sperm membrane integrity, chromatin structure, and functional competence [6], in agreement with studies documenting cisplatin-induced germ cell depletion and epididymal sperm dysfunction via apoptotic and oxidative DNA damage pathways [4,43].

A central finding of this work is that HES-PLGA-NPs delivery was associated with attenuated cisplatin-induced testicular damage, and that the magnitude of this effect was greater than that observed with free HES under the conditions tested. Nano-HES significantly restored testicular weight and improved sperm count, motility, viability, and morphology, suggesting effective rescue of spermatogenic activity and preservation of seminiferous tubule architecture. These protective effects are consistent with the known antioxidant and cytoprotective actions of HES, including suppression of ROS generation, inhibition of lipid peroxidation, and stabilization of mitochondrial function in germ cells [22,44]. HES has also been reported to support Sertoli cell function and protect germ cells against oxidative DNA fragmentation and apoptosis [21,23]. The more pronounced protective effects of the nanoformulation may be related to improved delivery characteristics (e.g., altered absorption, distribution, or release kinetics) rather than necessarily superior intrinsic biological activity; however, pharmacokinetic, biodistribution, and tissue accumulation studies are required to clarify the mechanisms underlying these enhanced effects [24,28]. Our in vitro data, including small, stable nanoparticles, high entrapment efficiency, and a sustained biphasic release profile, further support this interpretation.

Given that HES-PLGA-NPs were administered orally, it is relevant to consider how PLGA encapsulation may influence gastrointestinal stability and systemic exposure of hesperetin. Hydrophobic flavonoids, such as hesperetin, exhibit poor aqueous solubility and are prone to degradation and extensive first-pass metabolism in the gastrointestinal tract, resulting in modest oral bioavailability [45]. By encapsulating hesperetin within a PLGA matrix, the nanoparticles provide a physical barrier that protects the payload from acidic gastric conditions and luminal enzymes, improves apparent solubility, and enables controlled release in the intestinal environment [46]. PLGA-based nanocarriers have been shown in other models to enhance intestinal permeation and prolong residence time at the mucosal surface, thereby increasing systemic exposure and sustaining plasma levels of encapsulated drugs relative to their free forms [47,48]. In the present study, the more pronounced protective effects observed with oral HES-PLGA-NPs are therefore likely to reflect, at least in part, improved gastrointestinal stability and intestinal absorption of hesperetin, in addition to the local redox and signaling modulation documented at the testicular level. Nevertheless, dedicated pharmacokinetic and biodistribution studies will be required to quantify these contributions and to determine how oral nanoencapsulation reshapes hesperetin’s systemic disposition in cisplatin-treated animals.

Cisplatin also induced distinct endocrine disturbances, with significant reductions in serum testosterone, FSH, and LH. The decline in testosterone is consistent with Leydig cell dysfunction, mitochondrial impairment, and downregulation of steroidogenic enzymes such as StAR, CYP11A1, and 3β-HSD, which are highly sensitive to oxidative and inflammatory insults [10,49,50]. Concurrent reductions in FSH and LH likely reflect disruption of the hypothalamic–pituitary axis, possibly driven by systemic toxicity and neuroinflammation [51], ultimately impairing germ cell maturation, Sertoli cell support, and blood–testis barrier integrity [52]. Importantly, HES-PLGA-NPs normalized testosterone, FSH, and LH levels more effectively than free HES, with testosterone restored to near-control values. This suggests recovery at both gonadal and central regulatory levels. Previous studies have shown that HES protects Leydig cells by preventing mitochondrial dysfunction and attenuating cytokine-driven suppression of steroidogenic pathways [22,23,53]. Our findings extend these observations by suggesting that nanoencapsulation can enhance the endocrine benefits of HES in a clinically relevant cisplatin model, although the precise contribution of pharmacokinetics versus pharmacodynamics remains to be determined.

At the level of oxidative stress, cisplatin significantly increased MDA, NO, and 8-OHdG, indicating intensified lipid peroxidation, nitrosative stress, and oxidative DNA damage. Elevated MDA reflects peroxidation of polyunsaturated fatty acids in spermatozoa and testicular membranes [54], while excessive NO promotes peroxynitrite formation and damage to proteins and nucleic acids [55]. Higher 8-OHdG levels are a well-recognized marker of oxidative DNA damage linked to impaired fertility and germ cell loss [56]. These changes are consistent with cisplatin’s ability to drive ROS production through mitochondrial dysfunction, glutathione depletion, and NADPH oxidase activation [6,7]. In parallel, cisplatin markedly reduced GSH, SOD, and CAT, signifying collapse of the testicular antioxidant defense network. GSH depletion is particularly critical because GSH is indispensable for GPX4 activity, which detoxifies lipid hydroperoxides and links classical antioxidant defenses to ferroptosis [16,57]. Reduced SOD and CAT further exacerbate oxidative injury by impairing the dismutation of superoxide and breakdown of hydrogen peroxide [58].

Our data also suggest that cisplatin downregulated Nrf2/HO-1 signaling, a pivotal pathway for antioxidant gene induction and cytoprotection [59]. Under physiological conditions, oxidative stimuli promote Nrf2 nuclear translocation and upregulation of HO-1, GCLC, NQO1, and related enzymes [60], thereby enhancing cellular resilience to redox stress. The observed downregulation of Nrf2 and HO-1 in cisplatin-treated testes suggests a failure of adaptive antioxidant responses, consistent with recent reports on cisplatin-induced reprotoxicity [61,62]. In this context, HES-PLGA-NPs significantly attenuated oxidative stress, lowering MDA, NO, and 8-OHdG and restoring GSH, SOD, CAT, and GPx more effectively than free HES. They also reactivated Nrf2 and HO-1 at protein and transcript levels. These results agree with studies showing that HES and related flavanones activate Nrf2-dependent antioxidant programs and counteract chemotherapy-induced oxidative injury [19,63]. Taken together, the combination of direct ROS scavenging and Nrf2/HO-1 activation provides a plausible, though still correlative, mechanistic basis for the robust redox protection observed with nano-HES.

Inflammatory signaling was similarly amplified by cisplatin, as suggested by increased NF-κB expression and elevated TNF-α, IL-6, and IL-1β levels. NF-κB is a redox-sensitive transcription factor that integrates oxidative cues and injury signals to promote transcription of pro-inflammatory cytokines [64,65]. TNF-α and IL-1β disrupt blood–testis barrier integrity by altering tight junction proteins such as occludin and claudin-11, facilitating immune cell infiltration and exacerbating local inflammation [66]. IL-6 further impairs Sertoli cell function and germ cell adhesion, thereby compromising spermatogenesis [67]. The interplay between oxidative stress and NF-κB-driven inflammation creates a self-reinforcing cycle: ROS activates NF-κB, and, in turn, inflammatory cytokines generate additional ROS, a pattern frequently reported in cisplatin-related reproductive injury [12,13]. HES-PLGA-NPs effectively broke this cycle: they significantly suppressed NF-κB activation and reduced TNF-α, IL-6, and IL-1β, and again outperformed free HES. These results are congruent with evidence that HES mitigates inflammation by inhibiting NF-κB and dampening oxidative stress-triggered inflammatory cascades [21,23].

At the level of apoptosis, cisplatin increased Bax and caspase-3 while reducing Bcl-2, thereby elevating the Bax/Bcl-2 ratio and promoting mitochondrial membrane permeabilization and intrinsic apoptotic signaling. Cisplatin-induced DNA damage, p53 activation, and cytochrome c release are key upstream events driving caspase-dependent germ cell apoptosis [68]. Caspase-3 functions as the main executioner protease leading to DNA fragmentation and cell death [69], whereas reduced Bcl-2 diminishes mitochondrial resistance to oxidative and apoptotic insults [70]. In our study, HES-PLGA-NPs significantly attenuated these changes, lowering Bax and caspase-3 and increasing Bcl-2 levels more effectively than free HES. These data support the concept that nano-HES may preserve mitochondrial integrity and limit oxidative DNA injury, but they remain associative and do not demonstrate that modulation of these apoptotic markers is causally required for protection [22].

Beyond apoptosis, our results suggest that ferroptosis may contribute to cisplatin-induced testicular injury. Cisplatin downregulated GPX4 and SLC7A11 and upregulated ACSL4, while increasing Fe^2+^ accumulation, lipid ROS, and 4-HNE. GPX4 is the key lipid peroxidase that reduces phospholipid hydroperoxides to alcohols; its inhibition or depletion permits unchecked lipid peroxidation and ferroptotic cell death [16,57]. SLC7A11 mediates cystine uptake for GSH synthesis; its downregulation limits GSH availability and impairs GPX4 function, further driving ferroptosis [71]. ACSL4 enriches membranes with polyunsaturated fatty acyl chains, increasing their susceptibility to peroxidation [72]. Elevated Fe^2+^ enhances Fenton chemistry and ROS generation, while accumulation of 4-HNE reflects severe lipid peroxidation and membrane damage [72].

HES-PLGA-NPs could significantly counteract these ferroptosis-associated alterations, as these forms were associated with GPX4 and SLC7A11 upregulation and ACSL4 downregulation accompanied by reductions in Fe^2+^, lipid-derived ROS, and 4-HNE. These changes are consistent with attenuation of iron-dependent lipid peroxidation and restoration of glutathione-linked antioxidant defenses; however, definitive mechanistic confirmation of ferroptosis inhibition was not established because ferroptosis-specific inhibitors or complementary functional assays were not employed, and causal involvement of these pathways remains to be tested. Our findings align with emerging evidence that flavonoids, including HES derivatives, may modulate the Nrf2/GPX4/SLC7A11 axis and attenuate ferroptosis in chemotherapy-related tissue injury [73,74]. Taken together, the coordinated modulation of oxidative stress, NF-κB-mediated inflammation, apoptosis, and ferroptosis-associated markers may contribute to the tissue-protective and functional benefits observed with nano-HES. Future studies should incorporate ferroptosis-specific inhibitors, such as ferrostatin-1 or liproxstatin-1, and complementary functional assays to confirm the causal involvement of ferroptosis in cisplatin-induced testicular injury. Additional evaluation of mitochondrial morphology, labile iron pools, lipid peroxidation, and broader ferroptosis-related markers would further strengthen mechanistic validation.

Histopathological and immunohistochemical data corroborate the biochemical and molecular findings. Cisplatin caused extensive degeneration of seminiferous tubules, loss of germinal epithelium, vacuolization, and interstitial edema, along with reduced GPX4 and enhanced ACSL4 expression, consistent with ferroptosis-associated structural damage. In contrast, HES-PLGA-NPs preserved seminiferous tubule integrity, maintained organized germinal epithelium, and supported active spermatogenesis. They also restored strong GPX4 immunoreactivity and suppressed ACSL4 staining to near-control levels, providing visual evidence of ferroptosis inhibition and spermatogenic recovery.

**Figure 12:**
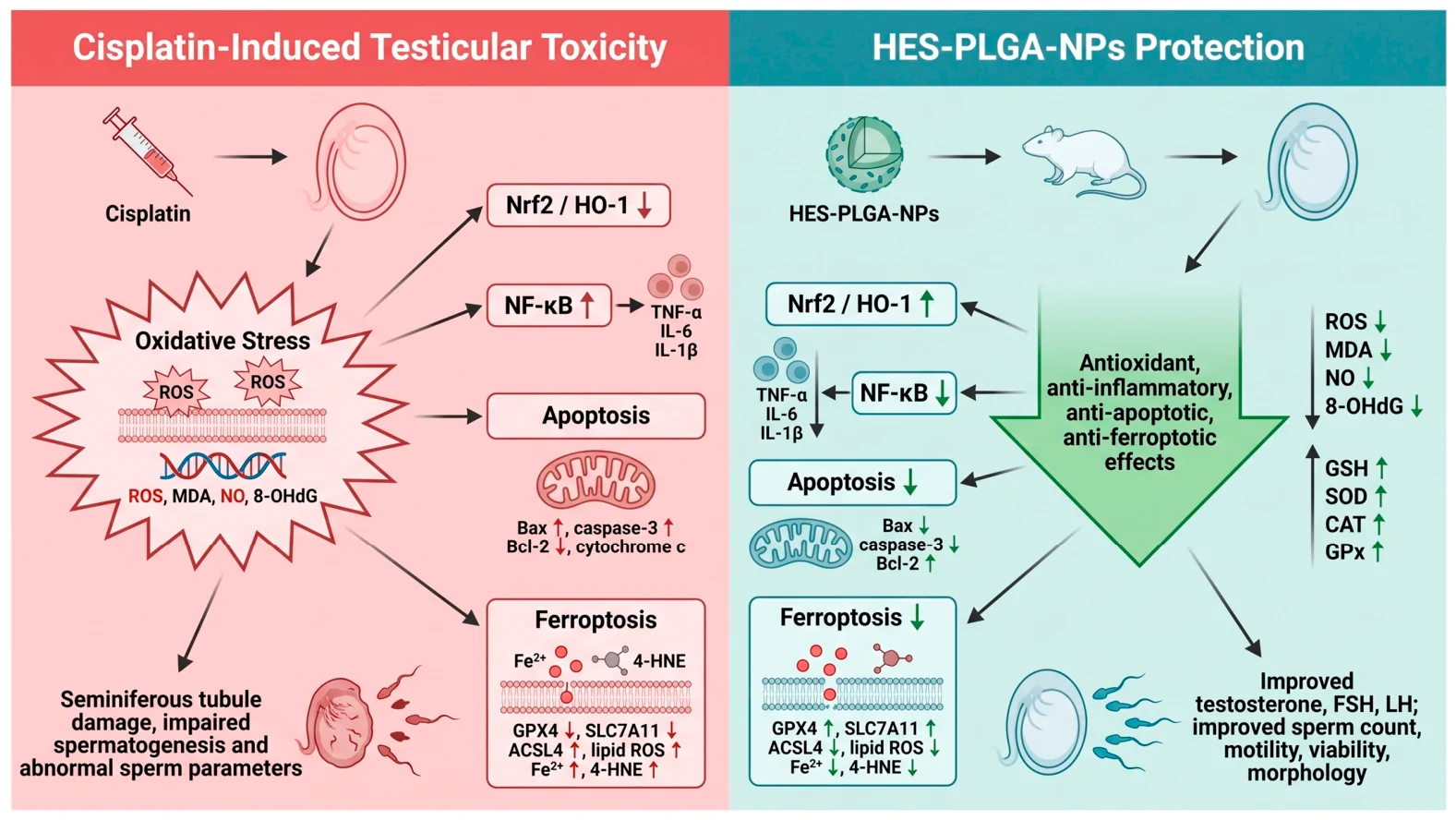
Proposed molecular mechanism by which hesperetin-loaded PLGA nanoparticles (HES-PLGA-NPs) ameliorate cisplatin-induced testicular toxicity. Cisplatin treatment enhances reactive oxygen and nitrogen species (ROS/RNS), lipid peroxidation (MDA, 4-HNE, lipid ROS), and oxidative DNA damage (8-OHdG), leading to depletion of antioxidant defenses (GSH, SOD, CAT, GPx) and downregulation of Nrf2/HO-1 signaling. These changes activate NF-κB-mediated inflammatory pathways (TNF-α, IL-6, IL-1β), promote mitochondrial apoptosis (↑ Bax, ↑ caspase-3, ↓ Bcl-2), and favor ferroptosis through Fe^2+^ accumulation, ACSL4 upregulation, and suppression of the SLC7A11–GSH–GPX4 axis. Oral administration of HES-PLGA-NPs is associated with restoration of Nrf2/HO-1 activity, normalization of antioxidant status, attenuation of NF-κB-driven inflammation and apoptosis, and modulation of ferroptosis-related markers (↑ GPX4/SLC7A11, ↓ ACSL4, ↓ Fe^2+^, ↓ lipid ROS), collectively contributing to preservation of spermatogenesis, testicular structure, and reproductive endocrine function. Generated with the assistance of the open source GAABSTRACT (https://gaabstract.com/).

The present results complement and extend earlier work on citrus flavonoids and hesperetin in reproductive toxicity models [23,24,29,75]. Building on that evidence base, our data now suggest that hesperetin-loaded PLGA nanoparticles confer better protection compared with free hesperetin in a cisplatin gonadotoxicity model and, importantly, that this benefit is accompanied by coordinated modulation of NRF2/HO-1, NF-κB, and ACSL4/GPX4/SLC7A11 ferroptotic signaling (Figure 12). By integrating nano-enhanced delivery with in vivo multi-pathway mechanistic analysis, the current study moves beyond earlier reports that considered these elements separately and provides a more comprehensive framework for understanding hesperetin-based strategies against chemotherapy-induced testicular dysfunction.

More broadly, these findings can be situated within the expanding field of flavonoid-loaded nanoparticles and ferroptosis-targeted therapies. PLGA nanoparticles loaded with hesperidin or hesperetin have been reported to enhance pro-apoptotic and cytotoxic effects in tumor cells [76,77,78], while several nanomedicine strategies specifically aim to induce ferroptosis in cancer cells or, conversely, to suppress it pharmacologically in non-malignant tissue using agents such as ferrostatin-1 and liproxstatin-1 [79,80,81]. The present work extends this literature in a complementary direction, showing that an oral hesperetin nanoformulation can attenuate ferroptosis-associated injury in normal testicular tissue during cisplatin chemotherapy, illustrating a tissue-protective application of flavonoid nanocarriers alongside their more commonly studied tumor-directed use [82].

Because cisplatin is an anticancer agent, an important translational consideration is whether antioxidant nanoparticle co-treatment might attenuate its antitumor efficacy. Cisplatin’s cytotoxicity in tumor cells involves ROS generation, DNA cross-linking, and apoptosis, and there has been longstanding concern that systemic antioxidants could theoretically blunt these effects [83]. However, multiple preclinical studies have reported that natural antioxidants and flavonoids, including hesperidin and hesperetin, not only protect normal tissues from cisplatin toxicity but can also enhance cisplatin’s antitumor activity by promoting cancer cell apoptosis, modulating PI3K/Akt and MAPK signaling, and improving chemosensitivity [19,84]. In the present study, we used non-tumor-bearing rats and did not evaluate tumor growth or cisplatin response; thus, we cannot draw conclusions about the impact of HES-PLGA-NPs on antitumor efficacy. Future work in appropriate tumor models will be essential to determine whether nano-hesperetin preserves or augments cisplatin’s anticancer activity while protecting male reproductive function, and to define dosing and scheduling strategies that avoid any compromise in oncological outcomes.

From a translational perspective, the current findings suggest that nano-HES may serve as a promising adjuvant strategy to protect male reproductive function in patients receiving cisplatin. The ability of HES-PLGA-NPs to simultaneously normalize reproductive hormones, improve sperm quality, and modulate oxidative stress, inflammation, apoptosis, and ferroptosis aligns with the multifactorial nature of chemotherapy-induced gonadotoxicity and the current interest in multi-target antioxidant approaches in male reproductive health [61,85]. Further work addressing pharmacokinetics, long-term safety, and potential interactions with antitumor efficacy, as well as formal causality tests using pharmacological and genetic modulation of key pathways, will be essential before clinical translation. However, the current data provide a strong experimental rationale.

### Limitations and Future Directions

The present work has several limitations that should be considered when interpreting the findings and planning subsequent studies. First, experiments were conducted in a single animal model, adult male rats, using a single cisplatin regimen and one dose level of HES and HES-PLGA-NPs. This design does not capture inter-species differences in cisplatin pharmacokinetics and testicular sensitivity, strain-specific variability, or the diversity of clinical chemotherapy protocols, age-related differences, and inter-individual variability in testicular susceptibility to gonadotoxicity. Because dose–response relationships and species-specific toxicodynamics were not evaluated, the optimal dose and schedule for HES-PLGA-NPs, as well as the generalizability of the present findings beyond this single rat model, remain to be established. Future studies should incorporate multiple dosing schedules, dose–response analyses, additional age groups, and, where feasible, complementary animal models or species to better approximate clinical scenarios and to confirm the robustness of the protective effects observed here. Second, we focused on short- to medium-term structural and functional endpoints and did not assess long-term fertility outcomes, such as mating performance, litter size, or potential transgenerational effects. These parameters are critical for translating testicular protection into clinically meaningful preservation of male reproductive capacity and warrant dedicated investigation in follow-up studies. Third, the experiments were performed in non-tumor-bearing animals; therefore, possible interactions between nano-hesperetin and the antitumor efficacy of cisplatin were not evaluated. Studies in appropriate tumor models will be essential to confirm that reproductive protection does not compromise oncological outcomes or alter cisplatin pharmacodynamics. Fourth, although we characterized key pathways related to oxidative stress, Nrf2/HO-1 signaling, NF-κB-mediated inflammation, apoptosis, and ferroptosis, other mechanisms implicated in cisplatin-induced testicular injury, such as endoplasmic reticulum stress, autophagy, cuproptosis, and HMGB1/TLR4 signaling, were not explored and remain important avenues for future mechanistic work. In addition, Western blot validation of the ELISA and immunohistochemical data was not performed because this technique was not available at our research facility; future studies should include Western blotting and additional molecular assays to strengthen protein-level confirmation. Moreover, the mechanistic insights presented here are based on associative changes in pathway markers rather than formal causality tests, and future studies employing pharmacological and genetic modulation of Nrf2/HO-1, NF-κB, and ferroptosis-related targets will be necessary to establish whether these pathways are required for nano-hesperetin-mediated protection. Fifth, the nanoparticle formulation was characterized for morphology, size and polydispersity, zeta potential, encapsulation efficiency at the time of preparation, FTIR profile, and in vitro release at physiological pH, but pharmacokinetic, biodistribution, and tissue accumulation studies were not conducted. Consequently, the more pronounced protective effects of HES-PLGA-NPs observed here should not be interpreted as definitive evidence of superior intrinsic bioactivity or improved bioavailability without supporting PK data. Moreover, long-term colloidal stability, quantitative drug loading, encapsulation efficiency after storage, and batch-to-batch reproducibility of HES-PLGA-NPs were not evaluated in this work and should be addressed in future formulation-focused studies to further substantiate the robustness and translational potential of this nanoformulation. Finally, we examined a single hesperetin nanoformulation. Systematic comparative studies with other nanoantioxidants and delivery platforms, extended dose–response analyses, and comprehensive safety profiling (including off-target organ toxicity and immunogenicity) will be important to optimize nano-HES and to define its place among emerging fertility-preservation strategies for male cancer patients.

## 5. Conclusions

In summary, cisplatin administration was associated with severe testicular toxicity in this rat model, characterized by endocrine disruption, impaired spermatogenesis, oxidative and nitrosative stress, inflammatory activation, apoptosis-related changes, and ferroptosis-associated alterations, culminating in marked structural and functional damage to the testes. Treatment with both HES and its PLGA-based nanoformulation was associated with significant attenuation of these alterations under the present experimental conditions, with HES-PLGA-NPs consistently showing a greater degree of attenuation than free HES.

HES-PLGA-NPs administration was associated with more pronounced restoration of serum testosterone, FSH, and LH, improved sperm quality and testicular weight, and higher levels of antioxidant markers, including Nrf2/HO-1 and classical enzymatic antioxidants, compared with free HES. It was likewise associated with lower NF-κB-related inflammatory markers, apoptotic markers, and ferroptosis-associated markers (ACSL4/GPX4/SLC7A11, Fe^2+^, lipid ROS, 4-HNE), alongside better-preserved seminiferous tubule architecture and active spermatogenesis. These findings indicate that HES-PLGA-NPs were associated with greater protection than free HES under the specific conditions of this study; however, this conclusion is based on associative biochemical, molecular, and histological endpoints rather than formal causality testing (e.g., pathway-specific inhibitors, knockdown models, or in vitro mechanistic assays), and pharmacokinetic, biodistribution, and tissue accumulation studies are required to elucidate the mechanisms underlying this enhanced efficacy.

Collectively, these preclinical findings identify HES-PLGA-NPs as a promising nanoantioxidant candidate warranting further investigation to mitigate cisplatin-induced testicular injury and support male reproductive function during chemotherapy. Given that the present study was conducted in non-tumor-bearing rats over a relatively short time frame and relied on associative rather than mechanistic evidence, its therapeutic potential remains to be established. It should not be assumed to translate directly to clinical use. Future studies incorporating pathway-specific mechanistic experiments (e.g., ferroptosis inhibitors, pharmacological or genetic modulation of Nrf2/NF-κB/ferroptosis targets), as well as pharmacokinetic, biodistribution, long-term fertility, tumor-model, and comprehensive safety evaluations, are essential before the therapeutic potential of HES-PLGA-NPs can be established for clinical application.

## Figures and Tables

**Table 1:** Primer sequences of target genes for RT-qPCR (antioxidant, inflammatory, and ferroptosis-associated markers).

Gene	Sequences (5′–3′)	Accession No.	Length (bp)
*Nrf2*	F: TTTGTAGATGACCATGAGTC	NM_031789.3	161
	R: TCCTGCCAAACTTGCTCCAT		
*NF-κB*	F: AGTCCCGCCCCTTCTAAAAC	NM_001415012.1	106
	R: CAATGGCCTCTGTGTAGCCC		
*GPX4*	F: ATTCGCAGCCAAGGACATCG	NM_017165.4	179
	R: TGCAAGGGAAGGCCAGGATT		
*ACSL4*	F: CCATATCGCTCTGTCACGC ACTTC	NM_001431649.1	118
	R: CCAGGCTGTCCTTCTTCCCAAAC		
*β-Actin*	F: CAGCCTTCCTTCTTGGGTATG	NM_031144.3	360
	R: AGCTCAGTAACAGTCCGCCT		

*Nrf2*, nuclear factor erythroid 2-related factor 2; *NF-κB*, nuclear factor kappa B; *GPX4*, glutathione peroxidase 4; *ACSL4*, acyl-CoA synthetase long-chain family member 4; *β-Actin*, beta-actin.

**Table 2:** Effect of HES and HES-PLGA-NPs on testicular weight and semen quality parameters in cisplatin-induced testicular toxicity.

Groups	Testicular Weight (g)	Sperm Concentration (×10^6^/Right Cauda Epididymis)	Progressive Sperm Motility (%)	Morphologically Abnormal Spermatozoa (%)	Sperm Viability (%)
Control	3.46 ± 0.09	80.44 ± 7.11	76.91 ± 5.99	5.63 ± 0.39	79.62 ± 7.14
HES	3.47 ± 0.10	80.78 ± 5.97	77.09 ± 6.24	5.21 ± 0.28	80.10 ± 8.61
HES-PLGA-NPs	3.49 ± 0.13	81.03 ± 7.15	78.14 ± 4.18	5.11 ± 0.47	80.27 ± 5.16
CIS	2.47 ± 0.11 *	46.39 ± 6.44 *	40.91 ± 4.72 *	16.24 ± 0.86 *	48.62 ± 4.77 *
CIS/HES	2.91 ± 0.05 *#	63.27 ± 4.87 *#	57.21 ± 5.37 *#	13.24 ± 0.75 *#	68.61 ± 4.12 *#
CIS/HES-PLGA-NPs	3.27 ± 0.03 *#+	71.89 ± 5.62 #+	69.46 ± 5.97 #+	10.78 ± 0.74 *#+	74.66 ± 5.42 #
*p*-values	0.0007	<0.0001	0.0001	<0.0001	<0.0001

Results are shown as mean ± SEM; Different symbols denote significant differences (*p* < 0.05) versus control (*), CIS (#), and free HES (+). Abbreviations: HES, hesperetin; HES-PLGA-NPs, hesperetin-loaded poly (lactic-co-glycolic acid) nanoparticles; CIS, cisplatin.

## Data Availability

The original contributions presented in this study are included in the article/Supplementary Material. Further inquiries can be directed to the corresponding author.

## Supplementary Materials

The following supporting information can be downloaded at: https://www.mdpi.com/article/10.3390/antiox15081007/s1, Table S1: Commercial kits, reagents, antibodies, fluorescent probes, and key instruments used in the study.

## Abbreviations

The following abbreviations are used in this manuscript:

2^−ΔΔCt^	Relative fold change calculated by the ΔΔCt method
4-HNE	4-Hydroxynonenal
8-OHdG	8-Hydroxy-2′-deoxyguanosine
ACSL4	Acyl-CoA synthetase long-chain family member 4
ANOVA	Analysis of variance
Bax	Bcl-2-associated X protein
Bcl-2	B-cell lymphoma-2
BM	Basement membrane
BSA	Bovine serum albumin
CAT	Catalase
CIS	Cisplatin
DAB	3,3′-Diaminobenzidine
DCM	Dichloromethane
DLS	Dynamic light scattering
DNA	Deoxyribonucleic acid
EE	Entrapment efficiency
ELISA	Enzyme-linked immunosorbent assay
FSH	Follicle-stimulating hormone
FTIR	Fourier transform infrared spectroscopy
GCLC	Glutamate–cysteine ligase catalytic subunit (mentioned in discussion)
GPx	Glutathione peroxidase
GPX4	Glutathione peroxidase 4
GSH	Reduced glutathione
H&E	Hematoxylin and eosin
HES	Hesperetin
HES-PLGA-NPs	Hesperetin-loaded poly(lactic-co-glycolic acid) nanoparticles
HO-1	Heme oxygenase-1
IL-1β	Interleukin-1 beta
IL-6	Interleukin-6
i.p.	Intraperitoneal
IHC	Immunohistochemistry
LH	Luteinizing hormone
MDA	Malondialdehyde
MWCO	Molecular weight cut-off
NF-κB	Nuclear factor kappa B
NO	Nitric oxide (measured as nitrite)
NQO1	NAD(P)H: quinone oxidoreductase 1 (mentioned in discussion)
Nrf2/NRF2	Nuclear factor erythroid 2-related factor 2
PBS	Phosphate-buffered saline
PDI	Polydispersity index
PLGA	Poly(lactic-co-glycolic acid)
PVA	Polyvinyl alcohol
qPCR	Quantitative polymerase chain reaction
ROS	Reactive oxygen species
SD	Standard deviation
SEM	Standard error of the mean
SLC7A11	Solute carrier family 7 member 11
SOD	Superoxide dismutase
SP	Spermatozoa
StAR	Steroidogenic acute regulatory protein (mentioned in discussion)
TEM	Transmission electron microscopy
TFR1	Transferrin receptor 1
TNF-α	Tumor necrosis factor-alpha
