# Supplementary material for: Hesperetin-Loaded PLGA Nanoparticles Ameliorate Cisplatin-Induced Oxidative Stress and Testicular Dysfunction in Rats: Association with NRF2/HO-1, NF-κB, and ACSL4/GPX4/SLC7A11 Signaling Modulation
Source: Antioxidants (Basel). 2026 Aug 13;15(8):1007. doi: 10.3390/antiox15081007 (PMC13509406; doi:10.3390/antiox15081007)
Supplement: Supplementary file 1 [file antioxidants-15-01007-s001.zip › antioxidants-4440489-supplementary.pdf]

Table S1. Commercial kits, reagents, antibodies, fluorescent probes, and key instruments used in the study

**Category**	**Item**	**Target / Use**	**Type**	**Catalog / Model**	**Manufacturer / Supplier**	**City, Country**	**Note**
Polymer	PLGA (50:50)	Matrix for HES-PLGA-NPs	Polymer, lactide: glycolide 50:50, acid-terminated	719870 (Resomer® RG 503 H; Mw 24,000–38,000)	Sigma-Aldrich (MilliporeSigma)	St. Louis, MO, USA	Used for nanoparticle synthesis
Stabilizer	Poly(vinyl alcohol) (PVA)	Emulsifier/stabilizer in nanoparticle preparation	Polymer, 87–89% hydrolyzed	363170 (Mw 13,000–23,000; 87–89% hydrolyzed)	Sigma-Aldrich (MilliporeSigma)	St. Louis, MO, USA	For primary/secondary emulsions
Chemical	Hesperetin (HES)	Active flavanone	≥98% purity	W431300	Sigma-Aldrich	St. Louis, MO, USA	Main Study target
Buffer	Phosphate-buffered saline (PBS), pH 7.4	Release medium, washes	Buffer powder	P5119	Sigma-Aldrich (MilliporeSigma)	St. Louis, MO, USA	For in vitro release and tissue handling
Reagent	Potassium bromide (KBr)	FTIR pellet preparation	FT-IR grade, ≥99%	221864	Sigma-Aldrich (MilliporeSigma)	St. Louis, MO, USA	For FTIR discs
Organic solvent	Acetone	Solvent for HES	HPLC grade	34850	Sigma-Aldrich (MilliporeSigma)	St. Louis, MO, USA	Used in organic phase
Organic solvent	Dichloromethane (DCM)	Solvent for PLGA	HPLC grade	34856	Sigma-Aldrich (MilliporeSigma)	St. Louis, MO, USA	Used in organic phase
Fixative	Neutral buffered formalin 10%	Testis fixation for histology/IHC	Ready-to-use	HT501640	Sigma-Aldrich (MilliporeSigma)	St. Louis, MO, USA	10% NBF
Anesthetic	Isoflurane	Anesthesia and euthanasia	Inhalational anesthetic	—	Fresenius Kabi	Bad Homburg, Germany	For induction and euthanasia
Drug	Cisplatin injection	Induction of testicular toxicity	Injectable solution	50 mg/50 mL vial	EIPICO (Egyptian International	10th of Ramadan City, Egypt	Provide strength and batch in text

					Pharmaceutical Industries Co.)		
Biochemical kit	TBARS/MDA assay kit	Malondialdehyde, lipid peroxidation	Colorimetric kit	MD 25 29	Bio-Diagnostic Co.	Giza, Egypt	Testicular homogenates
Biochemical kit	Nitric oxide (NO) assay kit	Nitrite (NO_2^-), NO production	Colorimetric (Griess)	NO 25 33	Bio-Diagnostic Co.	Giza, Egypt	Testicular homogenates
Biochemical kit	SOD activity kit	Superoxide dismutase	Colorimetric inhibition	SD 25 21	Bio-Diagnostic Co.	Giza, Egypt	Testicular homogenates
Biochemical kit	Catalase (CAT) activity kit	Catalase	Colorimetric	CA 25 17	Bio-Diagnostic Co.	Giza, Egypt	Testicular homogenates
Biochemical kit	GSH assay kit	Reduced glutathione	Colorimetric DTNB	GR 25 11	Bio-Diagnostic Co.	Giza, Egypt	Testicular homogenates
Protein assay	BCA protein assay	Total protein	Colorimetric kit (BCA)	23225 (Pierce™ BCA)	Thermo Fisher Scientific	Rockford, IL, USA	For normalization
ELISA kit	8-OHdG ELISA	8-Hydroxy-2′-deoxyguanosine	Rat ELISA	MBS165204	MyBioSource	San Diego, CA, USA	DNA oxidation in testis
ELISA kit	NRF2 ELISA	Nrf2	Rat ELISA	MBS3807961	MyBioSource	San Diego, CA, USA	Testicular homogenates
ELISA kit	HO-1 ELISA	HO-1	Rat ELISA	MBS2024438	MyBioSource	San Diego, CA, USA	Testicular homogenates
ELISA kit	NF-κB ELISA	NF-κB p65	Rat ELISA	MBS287521	MyBioSource	San Diego, CA, USA	Testicular homogenates
ELISA kit	TNF-α ELISA	TNF-α	Rat ELISA	AEES00516	Assay Genie	Dublin, Ireland	Testicular homogenates
ELISA kit	IL-6 ELISA	IL-6	Rat ELISA	RTES00015	Assay Genie	Dublin, Ireland	Testicular homogenates
ELISA kit	IL-1β ELISA	IL-1β	Rat ELISA	AEES02613	Assay Genie	Dublin, Ireland	Testicular homogenates
ELISA kit	Bax ELISA	Bax protein	Rat ELISA	MBS2512405	MyBioSource	San Diego, CA, USA	Apoptosis marker
ELISA kit	Bcl-2 ELISA	Bcl-2 protein	Rat ELISA	MBS457882	MyBioSource	San Diego, CA, USA	Anti-apoptotic marker

ELISA kit	Caspase-3 ELISA	Caspase-3	Rat ELISA	MBS743552	MyBioSource	San Diego, CA, USA	Apoptosis marker
ELISA kit	GPX4 ELISA	GPX4	Rat ELISA	EKU12535	Biomatik	Kitchener, ON, Canada	Ferroptosis marker
ELISA kit	4-HNE ELISA	4-Hydroxynonenal	Rat ELISA	MBS7606779	MyBioSource	San Diego, CA, USA	Lipid peroxidation marker
ELISA kit	ACSL4 ELISA	ACSL4	Rat ELISA	MBS8820647	MyBioSource	San Diego, CA, USA	Ferroptosis marker
ELISA kit	SLC7A11 ELISA	SLC7A11	Rat ELISA	MBS7204860	MyBioSource	San Diego, CA, USA	Ferroptosis marker
ELISA kit	Testosterone ELISA	Serum testosterone	Rat competitive ELISA	AEKE04981	Assay Genie	Dublin, Ireland	Serum hormones
ELISA kit	FSH ELISA	Follicle-stimulating hormone	Rat ELISA	CSB-E06869r	Cusabio	Wuhan, China	Serum hormones
ELISA kit	LH ELISA	Luteinizing hormone	Rat ELISA	CSB-E12654r	Cusabio	Wuhan, China	Serum hormones
Fluorescent probe	C11-BODIPY® 581/591	Lipid peroxidation (oxidized/reduced ratio)	Fluorescent probe	D3861	Thermo Fisher Scientific	Eugene, OR, USA	Testicular lipid peroxidation
Blocking reagent	Bovine serum albumin (BSA)	Blocking in IHC	Protein reagent	A3294	Sigma-Aldrich	St. Louis, MO, USA	5% BSA for blocking
Buffer	Citrate buffer (0.01 M, pH 6.0)	Antigen retrieval	Buffer	C9999	Sigma-Aldrich	St. Louis, MO, USA	For microwave retrieval
Reagent	Hydrogen peroxide solution	Endogenous peroxidase blocking	30% H_2O_2 stock	H1009	Sigma-Aldrich	St. Louis, MO, USA	Diluted to 3% in methanol
Chromogen	DAB Substrate Kit	HRP chromogen in IHC	DAB kit	ab64238	Abcam	Cambridge, UK	Brown precipitate
Detection reagent	Streptavidin–HRP	Signal development in IHC	Enzyme conjugate	ab7403	Abcam	Cambridge, UK	For biotinylated secondaries
Counterstain	Mayer's hematoxylin	Nuclear counterstain	Histology/IHC stain	MHS32	Sigma-Aldrich	St. Louis, MO, USA	For H&E and IHC

Stain	Hematoxylin and eosin (H&E)	General histology	Stains	H9627 (hematoxylin; E4382 (eosin Y)	Sigma-Aldrich	St. Louis, MO, USA	Routine histology
Stain	Eosin–Nigrosin	Sperm viability/morphology	Differential stain, laboratory-prepared	Eosin Y (230251), Nigrosin (43925)	Sigma-Aldrich	St. Louis, MO, USA	For live/dead and morphology
Antibody	Anti-GPX4 primary antibody	GPX4 (IHC)	Rabbit monoclonal	ab125066	Abcam	Cambridge, UK	Dilution 1:200; add RRID if available
Antibody	Anti-ACSL4 primary antibody	ACSL4 (IHC)	Rabbit monoclonal	ab155282	Abcam	Cambridge, UK	Dilution 1:200; add RRID if available
Antibody	Biotinylated goat anti-rabbit IgG H&L	Secondary for IHC	Biotin-conjugated secondary	ab6720	Abcam	Cambridge, UK	Used at 1:500
RNA kit	QIAamp RNeasy Mini Kit	Total RNA extraction from testis	Spin column kit	74106	Qiagen GmbH	Hilden, Germany	For RT-qPCR
Enzyme	RNase-free DNase I	On-column DNA removal	Enzyme	79254	Qiagen GmbH	Hilden, Germany	Optional cleanup
qPCR mix	SYBR Green One-Step RT-qPCR mix	RT and amplification	2× Master Mix	—	HERA, Willowfort	Birmingham, UK	Nrf2, NF-κB, GPX4, ACSL4, β-actin
qPCR enzyme	RT enzyme mix	Reverse transcriptase	20× mix	—	HERA, Willowfort	Birmingham, UK	Supplied with kit
Primers	Custom oligonucleotides	Nrf2, NF-κB, GPX4, ACSL4, β-actin	DNA primers	—	Metabion	Planegg/Steinkirche, Germany	Sequences in main Table 1 & primer sheet
nstrument	IKA C-MAG HS 7	Magnetic stirring/evaporation	Magnetic stirrer	C-MAG HS 7	IKA-Werke GmbH & Co. KG	Staufen, Germany	Nanoparticle preparation

Instrument	Bandelin Sonopuls HD 3200	Probe sonication	Ultrasonic homogenizer	HD 3200	Bandelin Electronic GmbH & Co. KG	Berlin, Germany	Emulsion size reduction
Instrument	Zetasizer Nano ZS	Particle size, PDI, zeta potential	DLS/ELS analyzer	ZEN3600	Malvern Panalytical	Malvern, UK	Nanoparticle characterization
Instrument	FTIR spectrometer	FTIR analysis	FTIR spectrometer	Spectrum Two	PerkinElmer	Waltham, MA, USA	HES, PLGA, HES-PLGA-NPs
Instrument	Rotary microtome	Paraffin sectioning	Microtome	Leica RM2235	Leica Biosystems	Nussloch, Germany	4–5 µm sections
Instrument	Light microscope	Histology and IHC	Upright microscope	Leica DM750	Leica Microsystems	Wetzlar, Germany	400× evaluation
Instrument	Refrigerated centrifuge	Sample centrifugation	Benchtop centrifuge	Universal 32R	Andreas Hettich GmbH	Tuttlinge, Germany	Nanoparticles and tissue spins
Instrument	StepOne™ Real-Time PCR System	RT-qPCR	Real-time PCR cycler	StepOne™	Applied Biosystems	Foster City, CA, USA	For ΔΔCt method
Software	SAS	Statistical analysis	SAS 9.4	SAS Institute Inc.	Cary, NC, USA	One-way ANOVA, Tukey HSD	
Software	GraphPad Prism	Graphing	Prism 9.1.0 (build 221)	GraphPad Software	San Diego, CA, USA	Figures and plots	
Software	ImageJ	IHC image analysis	Image analysis software	ImageJ v1.54	NIH	Bethesda, MD, USA	https://imagej.nih.gov
Software	StepOne™ Software	RT-qPCR data	Instrument software	—	Applied Biosystems	Foster City, CA, USA	Ct and melt curves
